# Supplementary material for: Are we really Bayesian? Probabilistic inference shows sub-optimal knowledge transfer
Source: PLoS Comput Biol. 2024 Jan 8;20(1):e1011769. doi: 10.1371/journal.pcbi.1011769 (PMC10798629; doi:10.1371/journal.pcbi.1011769)
Supplement: S1 Text — Analysing sensory weights learning across time in the learning phase. Handling arbitrarily large slopes: removal of larger than 1 slope values or logistic transformed slope. Outlier participant exclusion criteria. Supplementary results. Untransformed versus logistic transformed subject-specific prior values. Linear regression and likelihood-only modelled transfer scores. Simulated responses using the exemplar model. Supplementary references. (DOCX) [file pcbi.1011769.s001.docx]

**Supporting Text for**

**Are we really Bayesian? Probabilistic inference shows sub-optimal knowledge transfer.**

Authors: Chin-Hsuan Sophie Lin, Trang Thuy Do, Lee Unsworth, Marta I. Garrido

This file includes:

**Supplementary Methods**

**Analysing sensory weights learning across time in the learning phase**

**Handling arbitrarily large slopes: removal of larger than 1 slope values or logistic transformed slope**

**Outlier participant exclusion criteria**

**Supplementary results**

**Untransformed versus logistic transformed subject-specific prior values**

**Linear regression and likelihood-only modelled transfer scores**

**Simulated responses using the exemplar model.**

**Supplementary references**

**Supplementary Methods**

**Analysing sensory weight learning across time in the learning phase**

We want to know the time course of sensory weight learning to choose trials to be included for learning phase slope calculation. We acquired instantaneous slope by regressing the following ten trials (including the current trial) using the function polyfit.m in Matlab. The results are shown in **S1 Fig**.

**Handling arbitrarily large slopes: removal of larger than 1 slope values or logistic transformed slope**

We computed subject-specific priors using the eq.4 in the manuscript $\sigma_{Pi}^{2}= \frac{\sigma_{Li}^{2}*slope}{(1-slope)}$ . Occasionally slope values can be nearly identical to 1, which resulted in unrealistic negative prior variances (slope larger than 1) or very large estimates of prior variance (slope close to but smaller than 1). We evaluated two approaches managing close to 1 slope values. The first approach simply removed a larger than 1 slope for subject-specific prior estimation. In the second approach, we devised a “logistic-transformed” slope (1), namely replacing the slope in the eq.4 with logistic slope = (1/ (1+e^-slope^)) to restrict prior values between 0 to 1. Subject-specific priors acquired by this method are called transformed priors. We compared un-transformed versus transformed prior values. Results (supplementary results and **S2A Fig and S2B Fig**) showed that logistic transformation changed the relative order of priors between prior categories. We concluded that the first approach was more suitable for subject-specific prior and henceforth transfer score computations. It should be noted that slightly smaller than 1 slopes cannot be removed by the chosen method and still had potentials resulting in arbitrarily large transfer scores. In the next section (outlier participant exclusion criteria – Transfer score), we detailed how we identified and removed outliers of transfer scores related to but not exclusive to, this caveat.

**Outlier participant exclusion criteria**

**Slope** The 3MAD criterion was used to identified outliners in each prior/likelihood combination. Participants presenting with outlier slopes in all combinations used for the transfer phase were excluded from grouped analyses. The remaining and removed participant numbers can be found in the **S2 Table**.

**Subject-specific prior and predicted slope** A small proportion of participants were removed because measured slope values larger than 1 (see the previous section: **Handling arbitrarily large slopes**). No further outlier removal was applied. The remaining and removed participant numbers can be found in the **S2 Table**.

**Transfer score** Outliers of transfer scores in the experiment 1 were chosen based on the 3MAD criterion, the same as the criterion for slope data. For the experiment 2, using the two prior/likelihood combinations in the learning phase, two transfer scores were acquired for each participant. We delineated a method to identify participants who had unusually big discrepancies between the two transfer scores as outliers. We assigned participant’s two transfer scores such that one transfer score represented the x value and the other represented the y value in a 2D Euclidean space**.** Each participant was sitting at a position in the space, meaning that the coordinate for participant *i* is (x_i_,y_i)_. We then computed how far away this position was from the line y=x using the “point_to_line_distance.m” (function in Matlab). This Euclidean distance quantifies how much the two transfer scores differ. Any participants from whom distances were more than 3MAD from grouped distance median were then removed for grouped analysis. The remaining and removed participant numbers can be found in **S2 Table**.

**Supplementary results**

**Untransformed versus logistic transformed subject-specific prior values**

While the logistic transformation kept the relation between different estimations of the same objective prior unchanged, the specific numeric values have changed (**S2A Fig**). Importantly, this transformation also changed the relation between estimations of different prior sets (**S2B Fig**). We concluded that it is preferable to use the un-transformed estimations (over the logistic transformed) in the computation of the transfer score.

There are cases where arbitrarily large un-transformed prior variances could bias transfer scores and the reasons are as follows. The denominator of the transfer score, [predicted slope_transfer_-slope_learned_], equals [$\frac{\sigma_{Pi}^{2}}{(\sigma_{Pi}^{2}+\sigma_{Li\_transfer}^{2})} - \frac{\sigma_{Pi}^{2}}{\left( \sigma_{Pi}^{2}+\sigma_{Li\_learned}^{2} \right)}]$. This term can be re-written as $\frac{{(\sigma}_{Li\_learned}^{2}-\sigma_{Li\_transfer}^{2})}{(\sigma_{Pi}^{2}+\sigma_{Li\_learned}^{2}+\sigma_{Li\_transfer}^{2})}$, showing that the transfer score can overshoot beyond 1 when an estimated prior variance is arbitrarily larger than the likelihood uncertainty (as the denominator becomes arbitrarily small). This is an unavoidable measurement noise caused by using untransformed prior variances to compute the transfer score (but the reason to do so has been explained in the previous paragraph).

We further examined the effect of overestimations of priors (hence more likely showing a larger than 1 transfer score) in our results. **S2B Fig and S2C Fig** showed that overestimation of priors was most visible in the extrapolation condition in experiment 2. This is unsurprising given that slope values in the learning phase of the extrapolation condition were closer to 1. It is important to keep in mind that overestimations of priors in the extrapolation group would have obscured, rather than cause, the observed difference of transfer scores between groups.

**Linear regression and likelihood-only modelled transfer scores**

We found that for experiment 1, the modelled transfer scores assuming both linear regression and likelihood-only models (**S4A Fig and S4B Fig**), were larger than 1 for both serial and parallel groups in the discovery and validation sets. This pattern was different from the experimental data. For experiment 2, the best fitted linear regression models had close to zero coefficients of priors, meaning the linear regression model in the experiment 2 was practically a likelihood-only model. This was proven by the fact that modelled slopes and transfer scores acquired from both models were identical. In **S4 Fig** we thus only present simulated results of the linear model because results of the likelihood-only model were the same. Similar to experimental observations, there was an interpolation benefit, i.e. a significantly higher transfer score in the interpolation than the extrapolation conditions **(S4C Fig discovery set and S4D Fig validation set).** However, modelled transfer scores of the extrapolation group were not significantly different from 0 (discovery set *p* = 0.94, BF_10_ = 0.076, validation set *p* = 0.97, BF_10_ = 0. 052), which was not in line with the experimental data.

**Simulated responses using the exemplar model.**

We used the exemplar model to simulate experimental responses. For experiment 2, modelled transfer scores of the discovery set were like experimental data when the sampled exemplar number was 5. That is, an interpolation benefit was observed (**S5C Fig**). However, opposite to experimental findings, there was noticeable over-reliance on priors (**S5A Fig**). Another feature dissimilar to the experimental data was that modelled transfer-new trials slopes were significantly higher than Bayesian model predictions in both interpolation and extrapolation groups (**S5C Fig C and S5D Fig**). When we increased the sampled exemplar number to 20, over-reliance on prior became less obvious but the interpolation benefit vanished. Critically, transfer effects of both groups became insignificant (**S5D Fig**). Modelled results of experiment 1 and experiment 2 validation set had patterns highly like modelled results of experiment 2 discovery set, showing over reliance on priors and transfer score patterns similar to the real data in the case of small *N* but not moderate *N* (data not presented). Together with BIC results presented in the manuscript, evidence did not support that people utilise an exemplar model to perform the coin task.

**Supplementary references**

1. Vilares I, Kording KP. Dopaminergic medication increases reliance on current information in Parkinson’s disease. Nat Hum Behav. 2017;1(8):1–7.
