# Supplementary figures and images for: Are we really Bayesian? Probabilistic inference shows sub-optimal knowledge transfer
Source: PLoS Comput Biol. 2024 Jan 8;20(1):e1011769. doi: 10.1371/journal.pcbi.1011769 (PMC10798629; doi:10.1371/journal.pcbi.1011769)

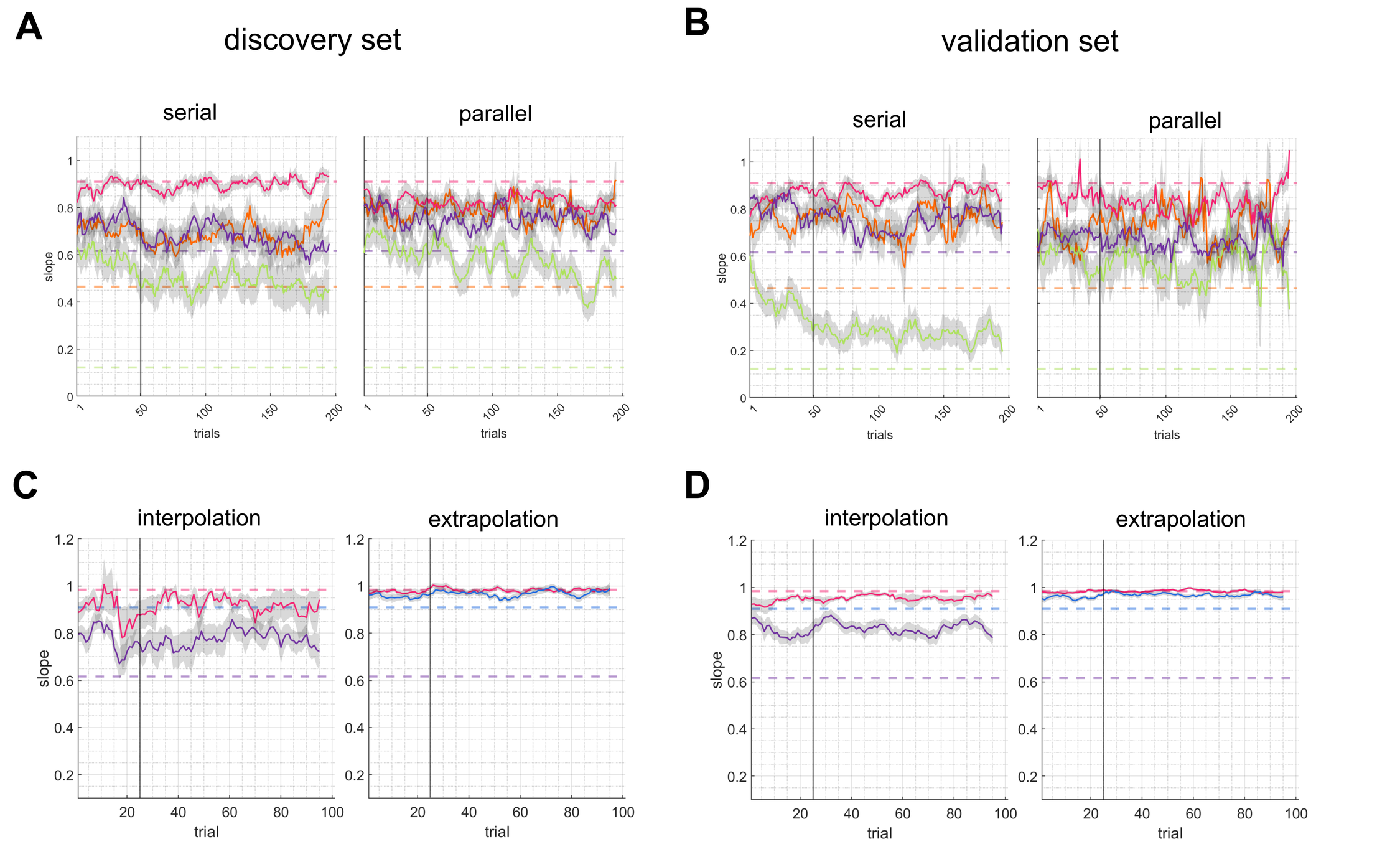

Supplement: S1 Fig — The instantaneous slope of each trial was calculated by regressing the following ten trials (including the current trial), except in trial 192–195 for experiment 1 and trial 92–95 for experiment 2 slope was computed by regressing the following six trials. The last 5 trials of each condition were not shown as to avoid biased slope presentations due to scarce data points. Coloured lines are group means and shades represent standard errors. The vertical line in each plot separates trials that were excluded (before)/included (after) for learning phase slope calculation in the main analysis. A discovery experiment 1 B validation experiment 1 C validation experiment 2 D validation experiment 2. (TIF) [file pcbi.1011769.s002.tif]

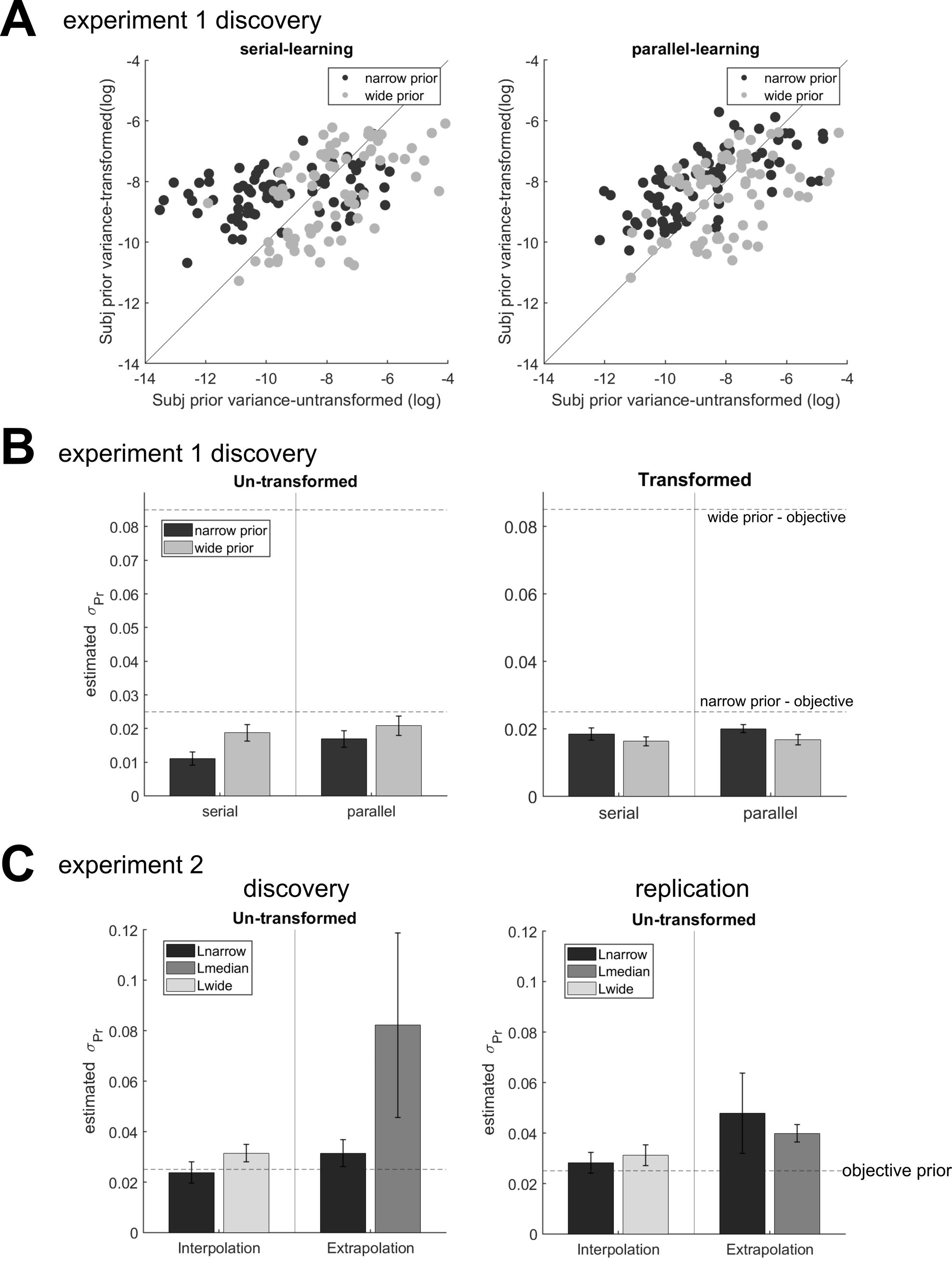

Supplement: S2 Fig — Subject-specific prior uncertainty (variance and standard deviation) A Scatter plots of prior estimates, experiment 1 discovery set. Left panel: serial learning group; right panel: parallel learning group. The X-axis represents untransformed prior and the Y-axis represents logistic-transformed prior. The order of estimates among the same experimenter designed prior categories are largely maintained after transformation but the distributions of values are compressed, as expected. B Bar figures to compare the group means of un-transformed (left panel) and transformed (right panel) subject-specific priors of experiment 1, discovery set. Error bars are standard errors. Dash lines are the experimenter designed standard deviations of narrow (= 0.025) and wide (= 0.085) priors. Figures show that the order of different priors only maintained in the untransformed estimations. They also show that the numerical values are generally smaller than the experimenter designed values. C Bar figures to compare the group means of un-transformed subject-specific priors of the experiment 2. Dash lines are the experimenter designed standard deviations of the prior (= 0.025). Overestimations were observed, especially in the extrapolation group. Left panel: discovery set; right panel: validation set. (TIF) [file pcbi.1011769.s003.tif]

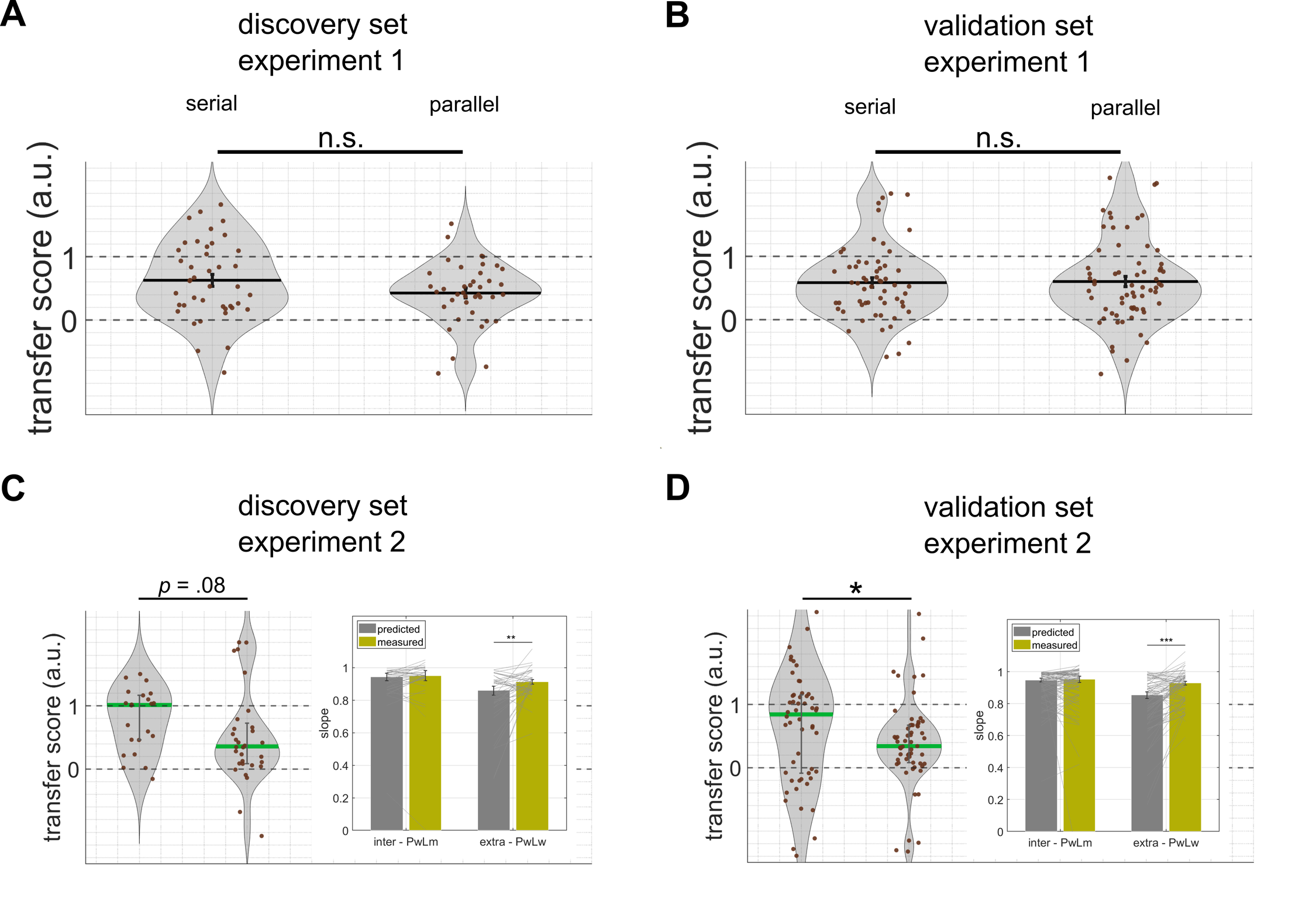

Supplement: S3 Fig — Violin plots show the distributions of transfer scores in A experiment 1 discovery set, B experiment 1 validation set, C experiment 2 discovery set, and D experiment 2 validation set. For A & B (experiment 1), the central black line is the mean of each group, and the vertical bar is the standard error. They closely resemble Fig 3C and 3D in the manuscript, showing that transfer scores largely locate between 0 and 1 and present no significant difference between serial and parallel groups. S3C and S3D Fig (experiment 2). The central green line is the median of each group, and the vertical bar is the interquartile range. They are also highly similar to Fig 4C and 4D in the manuscript, with the interpolation group showing higher transfer scores than the extrapolation group. Insets present predicted slope (grey bar) along with measured slope (olive bar) in the transfer-new trials. Note that for both discovery and validation sets, significant difference was only found in the extrapolation group. *p< = .05; ** p< = .01; ***p< = .001; n.s. non-significant. (TIF) [file pcbi.1011769.s004.tif]

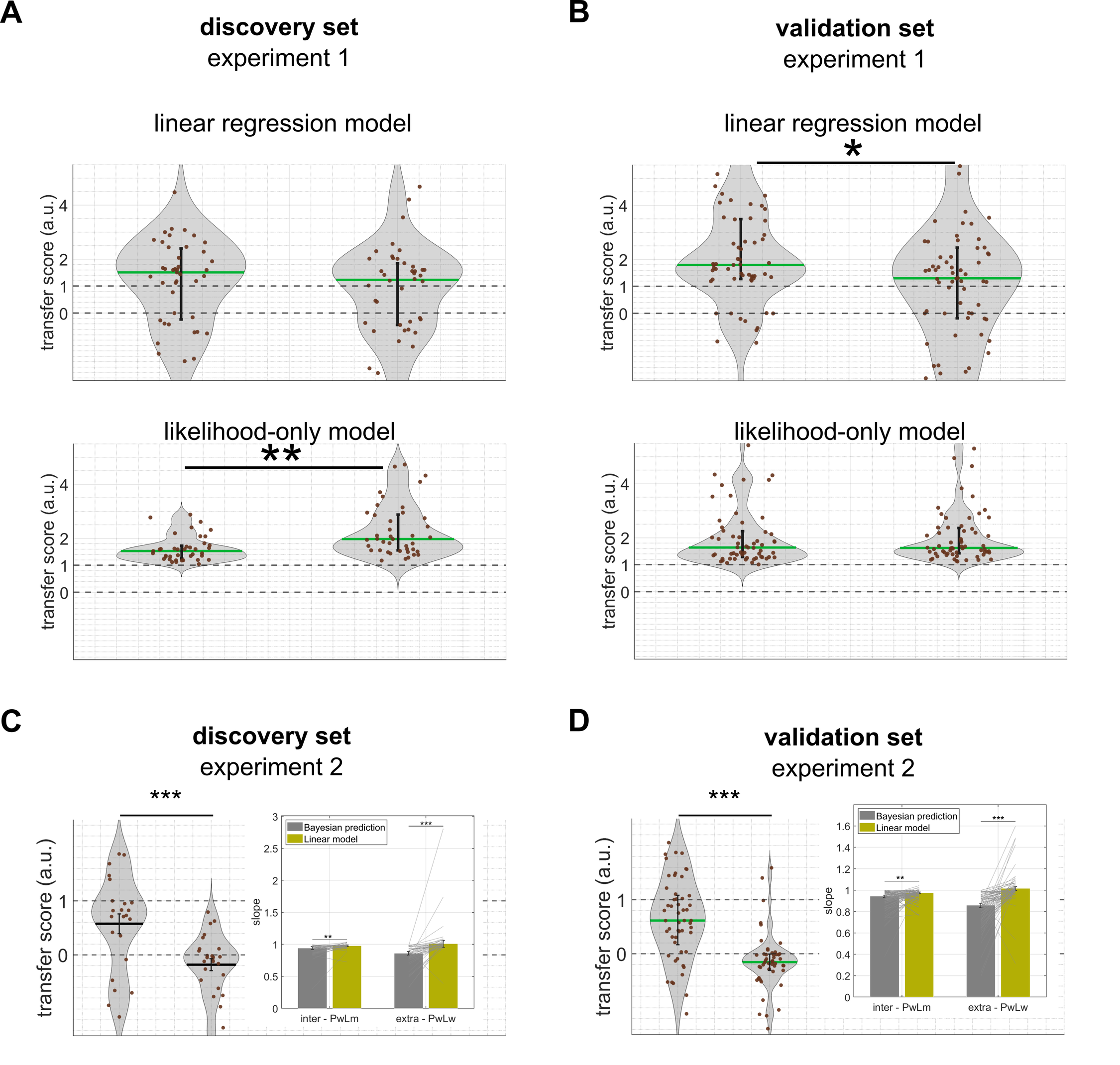

Supplement: S4 Fig — A (experiment 1 discovery set) & B (experiment 1 validation set) show modelled transfer scores. The central green line is the median of each group, and the vertical bar is the interquartile range. Transfer scores are larger than 1 for both discovery and validation sets. C (experiment 2 discovery set) & D (experiment 2 validation set) show modelled transfer scores and insets compare slopes predicted by Bayesian model (grey bar) along with linearly modelled slopes (olive bar) of the transfer-new trials. Two important features are found: (1) transfer scores of the interpolation group are higher than those of the extrapolation group and (2) transfer scores of the extrapolation group are not significantly different from 0. *p< = .05, **p< = .01, ***p< = .001; n.s. non-significant. (TIF) [file pcbi.1011769.s005.tif]

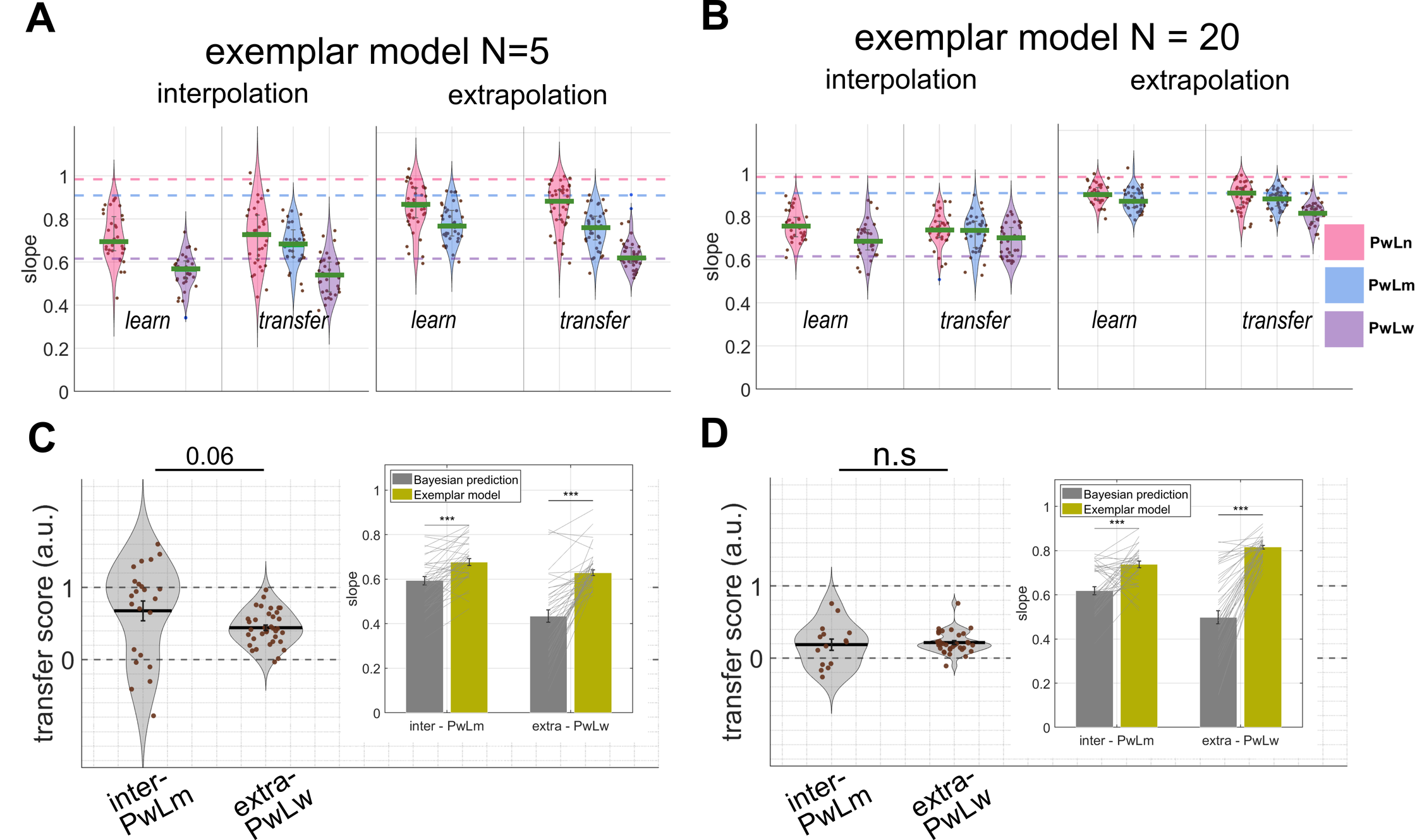

Supplement: S5 Fig — A (sampled exemplar size N = 5) & B (sampled exemplar size N = 20) show the distributions of modelled sensory weights and C (N = 5) & D (N = 20) show modelled transfer scores. Insets of C & D show predicted slope (grey bar) along with exemplar modelled slope (olive bar) of the transfer-new trials. ***p< = .001; n.s. non-significant. (TIF) [file pcbi.1011769.s006.tif]
