# Supplementary material for: Are we really Bayesian? Probabilistic inference shows sub-optimal knowledge transfer
Source: PLoS Comput Biol. 2024 Jan 8;20(1):e1011769. doi: 10.1371/journal.pcbi.1011769 (PMC10798629; doi:10.1371/journal.pcbi.1011769)
Supplement: S1 Table — (PDF) [file pcbi.1011769.s007.pdf]

**Experiment 1**		
	**discovery** (mean±SEM)	**validation** (mean±SEM)
PnLn	.0021±.0007	.0028±.0005
PnLw	.0016±.0008	.0036±.0009
PwLn	.0011±.0005	.0014±.0008
PwLw	.0046±.0013	.0043±.0009
**Experiment 2**		
	**discovery** (mean±SEM)	**validation** (mean±SEM)
PwLn	.0028±.0016	.0011±.0003
PwLm	.0024±.0005	.0025±.0004
PwLw	.0055±.0026	.0036±.0010
