# Supplementary material for: Are we really Bayesian? Probabilistic inference shows sub-optimal knowledge transfer
Source: PLoS Comput Biol. 2024 Jan 8;20(1):e1011769. doi: 10.1371/journal.pcbi.1011769 (PMC10798629; doi:10.1371/journal.pcbi.1011769)
Supplement: S2 Table — Numbers in brackets are outliers who were excluded based on the criteria described in the supplementary methods. (PDF) [file pcbi.1011769.s008.pdf]

participants N included(excluded)	**slope**	**Subject-specific prior/predicted slope**	**transfer score**
**Experiment 1**			
Discovery - serial	48(0)	44(3)	43(1)
Discovery - parallel	47(1)	47(0)	43(4)
Validation - serial	78(2)	72(6)	67(5)
Validation - parallel	78(1)	75(3)	66(9)
**Experiment 2**			
Discovery - interpolation	35(0)	33(2)	28(5)
Discovery - extrapolation	40(0)	39(1)	28(11)
Validation - interpolation	82(0)	79(3)	68(11)
Validation - extrapolation	82(0)	73(9)	59(6)
