# Supplementary material for: Are we really Bayesian? Probabilistic inference shows sub-optimal knowledge transfer
Source: PLoS Comput Biol. 2024 Jan 8;20(1):e1011769. doi: 10.1371/journal.pcbi.1011769 (PMC10798629; doi:10.1371/journal.pcbi.1011769)
Supplement: Attachment — Submitted filename: Response to reviewers.pdf [file pcbi.1011769.s009.pdf]

Are we really Bayesian? Probabilistic inference shows sub-optimal knowledge transfer

Response to reviewers

We thank the reviewers for their constructive comments. In the following, we respond to each point of these comments with texts in blue colour. We also marked changes to the manuscript text in **bold font** and provided their line numbers in the updated manuscript.

Reviewer #1:

Reviewer: Michael Landy

This paper is one among a few recent attempts to push on the idea that humans make full Bayesian calculations for perceptual estimation by using Mamassian/Maloney's idea of Bayesian transfer. This paper makes a stronger case for a weak, sub-optimal inference computation using a boatload of online data and indices that tailor the hypothesis test to individual observers. It is a significant addition to the literature and worthy of being published. I do have some comments, however none of them are hugely important.

Specifics, mostly by line number:

1. L117: For what it's worth, as far as I know, Konrad got the splatter of dots task, including the unseen coin dropped in the lake/you get to see the bubbles allegory from my paper with Tassinari and Hudson. Note that that paper also asked whether inference was precisely Bayesian, and indicated that it wasn't. We wrote our paper in response to Körding/Wolpert. There paper, cited a gazillion times, claimed that motor responses were Bayesian when, in fact, they FIT a Bayesian observer to the data (rather than measuring the likelihood and training a prior as you and we did).

Later on in the paper, especially in the Discussion, you point out that although Bayesian calculations in general can be quite complex, in a learning design with a fixed prior and likelihood, all Gaussian, then observers needn't do the Bayesian calculation at all and need only learn a weight. I was very happy with this overt discussion of the simplicity of the Gaussian case. That kind of observer doesn't know how to generalize and, without feedback, you'd expect they wouldn't. I like the idea that they might use this learned slope for a variety of conditions and reason toward a guessed slope for new conditions when the new condition is, well, inside the convex hull of what they've learned before (as you talk about in the Discussion). I sign all my reviews, so it's not inappropriate to mention that I've complained to Konrad in multiple reviews that claiming Bayesian when all that's needed is to learn a slope (i.e., do linear regression) is, well, unconvincing. I've also claimed that people seem indiscriminable from optimal in various contexts, but clearly that claim won't always hold!

Minor thought: The slope is constrained in a Bayesian calculation to lie between 0 and 1. First, that might result in range effects (i.e., estimates that avoid the ends of the range), but it's also true that data can result in slopes outside the range. I doubt this bears discussion in the paper, though. On the other hand, you report slopes from a linear regression with an intercept, i.e., you don't force the regression line to go through the center of the prior (i.e., the center of the display). Would doing so change any conclusions? Do you have evidence for intercepts significantly different from zero? (I hope not...). Note: your transfer score is about slopes that come out too low, but if you extrapolate to a prior that is narrow (something you didn't do in your experiment), a conservative (too small) extrapolation of slope would lead to a measured slope that was too big. Your transfer score, however, deals with that outcome correctly, given that you look at change in slope.

Thank you for the detailed and constructive feedback regarding the tasks and data analysis. Our replies to the suggestions (in the section "minor thoughts") are as follows:

a. Values of intercepts, i.e. prior mean

We examined the values of regression line intercepts. While we did find small biases, the values of these biases were much smaller than the width of the net (.02) used to make a response (mean±std; discovery experiment 1: 0.0023±0.0062, validation experiment 1: .0031±.0070, discovery experiment 2: .0033±.0125, validation experiment 2: .0021±.0056) Below we also include supplementary table S1, which we listed the group means of the biases of each prior/likelihood pairs) The findings of small offsets are consistent with a previous study (Sato & Körding, 2014) and the findings from our lab (Pike et al., in preparation) that are in keeping with a bias in the expectation of objects in space (pseudoneglect, see also (Garrido & Deouell, 2021)).

Here we also include supplementary **table S1,**

**Experiment 1**		
	**discovery** (mean±SEM)	**validation** (mean±SEM)
PnLn	.0021±.0007	.0028±.0005
PnLw	.0016±.0008	.0036±.0009
PwLn	.0011±.0005	.0014±.0008
PwLw	.0046±.0013	.0043±.0009
**Experiment 2**		
	**discovery** (mean±SEM)	**validation** (mean±SEM)
PwLn	.0028±.0016	.0011±.0003
PwLm	.0024±.0005	.0025±.0004
PwLw	.0055±.0026	.0036±.0010

To ensure these small biases had no significant effects on transfer behaviours, we repeated the calculations of transfer scores using slopes acquired when forcing intercepts of regression lines into zeros. We demonstrated that the observations and conclusions of the manuscript remain unchanged (**Figure S3**).

We explained the rationale of performing this additional analysis and findings of it in the manuscript (L528):
"In our data, fitted linear functions had minuscule non-zero intercepts (mean±std 0.0027±0.0078, see supplementary Table S1 for group means of each prior/likelihood combinations). Similar findings have been found (Sato & Körding, 2014 and E.Pike and M.I. Garrido unpublished observations) and these previous works showed small biases had no effects on Bayesian slopes. To ensure our results were unaffected by the bias, we computed transfer scores using slopes acquired when forcing the regression line intercept to zero (supplementary Figure S3). We confirmed that transfer behaviours in both experiments were not affected by non-zero intercepts."

Below we also include the **Figure S3** and its caption.

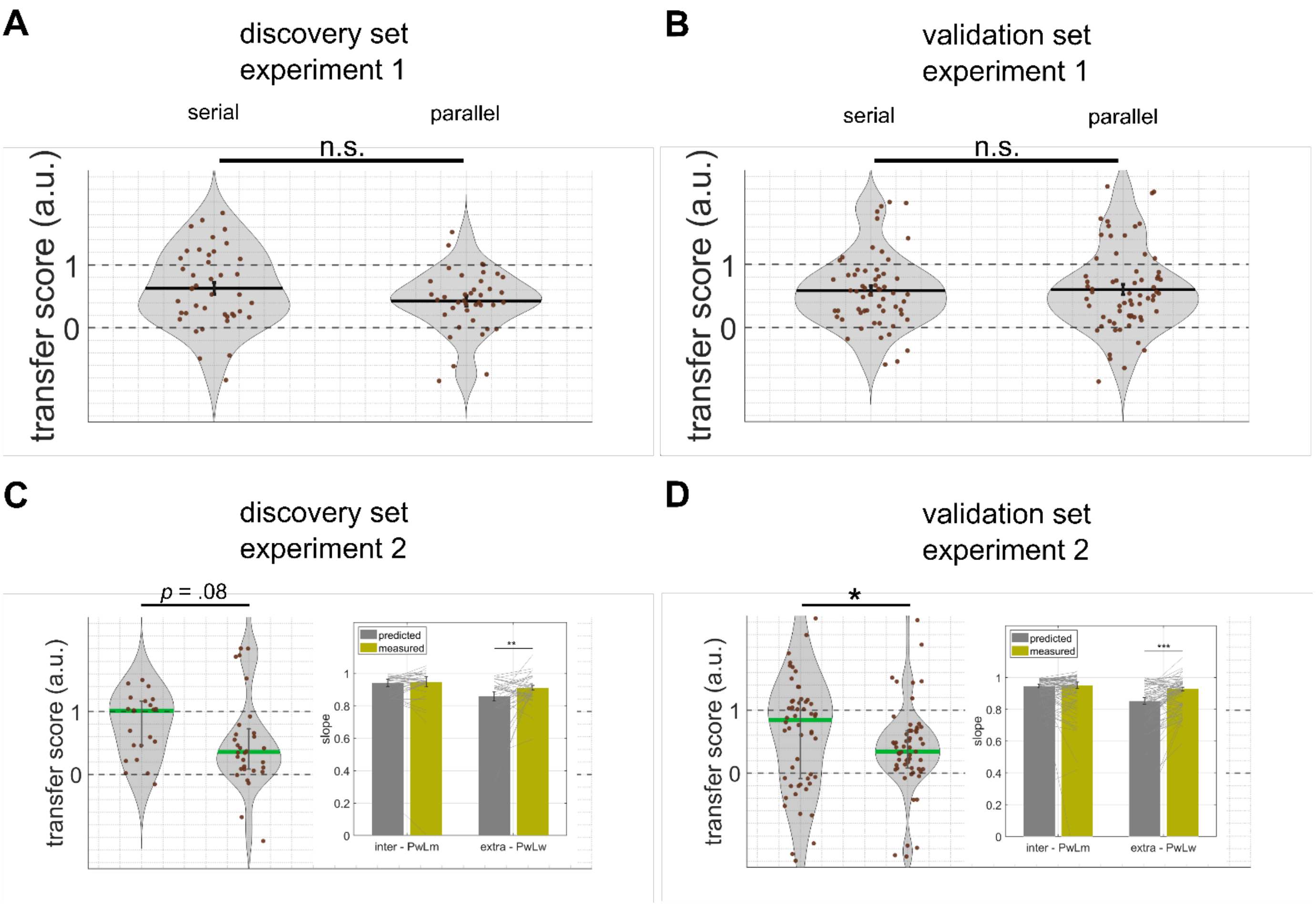

Figure S3 Transfer scores computed using slopes acquired when forcing the regression line intercept zero. *Violin plots show the distributions of transfer scores in* ***A*** *experiment 1 discovery set,* ***B*** *experiment 1 validation set,* ***C*** *experiment 2 discovery set, and* ***D*** *experiment 2 validation set. For* ***A & B*** *(experiment 1), the central black line is the mean of each group, and the vertical bar is the standard error. They closely resemble* ***Fig 3.C & D*** *in the manuscript, showing that transfer scores largely locate between 0 and 1 and present no significant difference between serial and parallel groups. For* ***C & D*** *(experiment 2), the central green line is the median of each group, and the vertical bar is the interquartile range. They are also highly similar to* ***Fig 4.C & D*** *in the manuscript, with the interpolation group showing higher transfer score than the extrapolation group. Insets present predicted slope (grey bar) along with measured slope (olive bar) in the transfer-new trials. Note that for both discovery and validation sets, significant difference was only found in the extrapolation group. *p<= .05; ** p<= .01; ***p<=.001; n.s. non-significant*

b. Transfer score

Thank you for pointing out that our transfer score compares ‘changes’ of slopes instead of slope value. To express it more explicitly in the manuscript, we included the following sentence in the ‘Methods-Data analysis-Transfer score’ section (L556): “**The transfer score compares the measured ‘change’ of slopes against a predicted slope change computed by subject-specific estimates of prior and likelihood**”.

2. L148 or so: I was surprised you see to calculate slope here from the entire learning phase, rather than just from later trials. You don't do anything to demonstrate learning here or measure its time course, or show that it has asymptoted by the end of the phase.

Thank you for pointing this out. We have now re-calculated the slopes of the learning phase. Specifically, we removed the first 50 trials of each prior in the learning phase (Note that for the experiment 2, this meant removing the first 25 trials of each prior/likelihood combination because there was only one prior.). The

number of trials removed was based on the time course of slopes of our data (supplementary **Figure S1**) and the original coin task paper (Vilares et al., 2012). We are pleased to report that our main results and conclusions remained unchanged. We have added this change to the Methods section (L522): "**We discarded the first 50 trials (experiment 1)/25 trials (experiment 2) of every prior/likelihood pair (based on ref** (Vilares et al., 2012) **and the findings of supplementary Figure S1) to minimize the effect of the initial learning phase."** and updated all the relevant numerical values of **Results**.

Here we also include **Figure S1** and its caption.

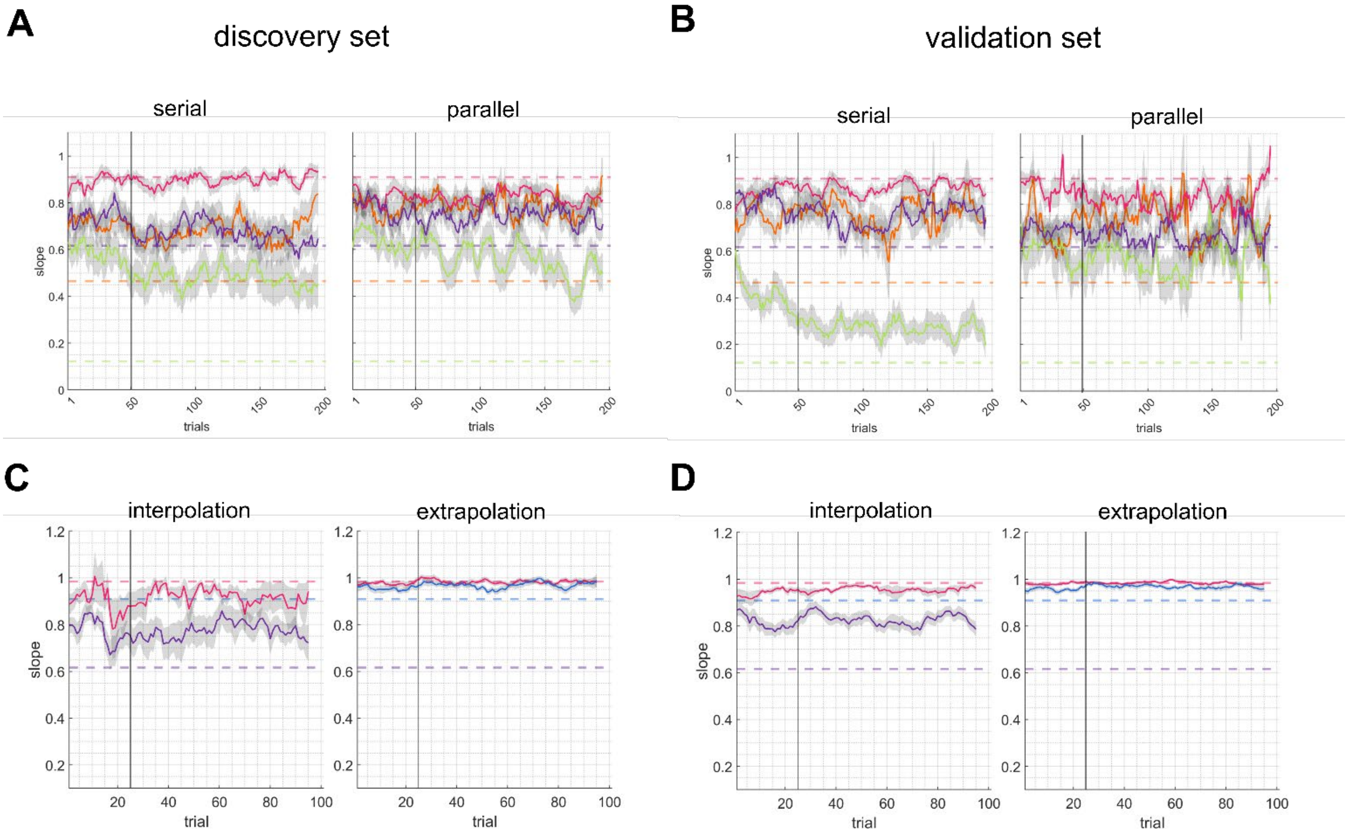

Figure S1. Time course of slopes in the learning phase. The instantaneous slope of each trial was calculated by regressing the following ten trials (including the current trial), except in trial 192-195 for experiment 1 and trial 92-95 for experiment 2 slope was computed by regressing the following six trials. The last 5 trials of each condition were not shown as to avoid biased slope presentations due to scarce data points. Coloured lines are group means and shades represent standard errors. The vertical line in each plot separates trials that were excluded (before)/included (after) for learning phase slope calculation in the main analysis. A discovery experiment 1 B validation experiment 1 C validation experiment 2 D validation experiment 2.

3. L166, 169: Here you are reporting non-significant effects (accepting the null) as evidence for the null. Please don't do that! Later, you do report a Bayesian test (which apparently doesn't show evidence for the null either), but here it's simply a no-no.

We have corrected both sentences to report null effect evidence as follows.

L177(previous L166) "Wilcoxon signed rank test *p* = .55, evidence of phase effect BF_{10} = .18"

L181(previous L169) "Model including prior and likelihood effect v.s. model including only prior effect: log likelihood ratio = 1.00, *p* = 0.36."

4. L240: "equivalent evidence" isn't a thing, is it? More like equivocal, or simply no evidence either way.

Thank you for pointing this out. The numerical value has changed after we recalculated the learning phase slopes and the sentence has been updated according to the new statistical result:

L250 (previous L240) "The t-test statistics is marginally significant (two side rank sum test *p* = 0.08)"

5. L256: What's "Bayesian-fashioned"? ;^)

We have replaced "Bayesian-fashioned" with "**qualitatively Bayesian**"**(L270)** in the manuscript.

6. L301:"its complex calculations" are not complex for this Gaussian case

We explained how complex calculations can be replaced with linear models for the Gaussian noise coin task in the discussion.

L355: "Due to the Gaussian noise feature of the coin task, the optimal policy is a linear mapping between stimuli and response (eq 1). We need to entertain the possibility that participants could have capitalised on this feature and learned a mapping for one stimulus type without forming a generative model."

7. Line 265 & 520: The optimality index is a bit odd. You say it's a trial-by-trial statistic, but you already demonstrate that the likelihood width is wider than simply the dot variance divided by the number of dots. Thus, there are additional sources of noise in observer responses, such as noisy measurement of dot positions, noisy calculation of the centroid, noisy calculation of the weighted average and/or noisy positioning of the net. Noise at the output (motor noise in positioning the net) would affect all likelihood/prior combinations and could affect your conclusions (we discuss an analogous issue with late noise in one of the optimal-cue-integration papers (Jamie Hillis with me, Marty Banks and others, in JOV). It would have been nice if you had a task to estimate the output noise, which is also involved in the likelihood-only task (i.e., that task is not only likelihood!). Also, on a given trial, you know where the centroid was, but you don't know what the internal centroid-location measurement was, so your estimate of optimality index is not, and can't be, based on the internal evidence the participant had to work with.

Thank you for raising this point. While we agree that there are multiple sources of noise affecting observers' responses, our purpose of using the optimality index was to understand if there were (unwanted) behavioural changes contingent upon the absence of coin position feedback in the transfer phase. The exact internal processes were not the focus of this analysis. For transfer-old trials, we wanted to confirm that performance level did not drop right after the disappearance of the feedback. For transfer-new trials, we wanted to know if there were any significant changes (improvement or decline) of performance between the beginning and the end of the transfer phase. Optimality index was chosen because it is (1) less susceptible to the effect of priors and likelihoods (2) more stable than slope on a trial-by-trial basis. We admit that we did not explicitly stated these purposes to our readers previously.

To clarify the goal of examining the optimality index, we have added a paragraph in the Methods (L571):

"We wanted to understand whether people's weighting of likelihood changed in the absence of feedback on the coin position during the transfer phase. For transfer-old trials, we wanted to confirm that the performance level did not drop right after the disappearance of the feedback. For transfer-new trials, we wanted to show that there were no significant changes (improvement or decline) of performance between the beginning and the end of the transfer phase. In this context, the multiple prior-likelihood combinations within and between participants became confounding factors. We therefore adopted a general measure of performance: the "optimality index", that is applicable beyond the same prior-likelihood combinations from (Acerbi et al., 2014)**."**

8. L484: Why the script L in the running text but not in the display equations (for likelihood variance)?

We have updated the text to ensure we use the symbol "σ_L^2" to represent likelihood variance throughout the manuscript.

9. L502/Eq. 2: This is subject-specific variance, but is NOT subjective variance (that would be the variance the subject has in his/her head, which could indeed differ from their actual variance).

We have updated the texts and equation 2 as the reviewer has suggested.

10. L505: I like the idea of inferring these parameters of the individual subjects for testing transfer. However, the formula here for the variance of the prior is a ratio and a bit complex and likely is typically a biased estimate, which is worth worrying about.

Thank you for your appreciation of our approach. We admit that because of the ratio nature, one of the biggest concerns is that the values can be biased by arbitrarily big prior estimates. We devoted multiple sections in the supplementary materials (supplementary Methods - Handling arbitrarily large slopes & Outlier participant exclusion criteria and **Figure S2**) to explain how we made our best efforts to mitigate the effects of arbitrarily large slopes on subject-specific prior and transfer score while not cherry-picking results favouring our conclusions.

The supplementary methods section - "**Handling arbitrarily large slopes: removal of larger than 1 slope values or logistic transformed slope"** is as follows: **"We computed subject-specific priors using the eq.3 in the manuscript $\sigma_{Psub}^2 = \frac{\sigma_{Lsub}^2 * slope}{(1-slope)}$. Occasionally slope values can be nearly identical to 1, which resulted in unrealistic negative prior variances (slope larger than 1) or very large estimates of prior variance (slope close to but smaller than 1). We evaluated two approaches managing close to 1 slope values. The first approach simply removed a larger than 1 slope for subject-specific prior estimation. In the second approach, we devised a "logistic-transformed" slope (1), namely replacing the slope in the eq.3 with logistic slope = (1/ (1+e^{-slope})) to restrict prior values between 0 to 1. Subject-specific priors acquired by this method are called transformed priors. We compared un-transformed versus transformed prior values. Results (supplementary results and Figure S2 A & B) showed that logistic transformation changed the relative order of priors between prior categories. We concluded that the first approach was more suitable for subject-specific prior and henceforth transfer score computations. It should be noted that slightly smaller than 1 slopes cannot be removed by the chosen method and still had potentials resulting in arbitrarily large transfer scores. In the next section (outlier participant exclusion criteria – transfer score), we detailed how we identified and removed outliers of transfer scores related to but not exclusive to, this caveat."**

And in the supplementary methods section - **Outlier participant exclusion criteria**, we explained that

"Outliers of transfer scores in experiment 1 were chosen based on the 3MAD criterion, the same as the criterion for slope data. For experiment 2, using the two prior/likelihood combinations in the learning phase, two transfer scores were acquired for each participant. We delineated a method to identify participants who had unusually big discrepancies between the two transfer scores as outliers. We assigned participant's two transfer scores such that one transfer score represented the x value and the other represented the y value in a 2D Euclidean space. Each participant was sitting at a position in the space, meaning that the coordinate for participant *i* is (x_i,y_i). We then computed how far away this position was from the line y=x using the "`point_to_line_distance.m`" (function in Matlab). This Euclidean distance quantifies how much the two transfer scores differ. Any participants from whom distances were more than 3MAD from grouped distance median were then removed for grouped analysis. The remaining and removed participant numbers can be found in table S2."

Figure S2 and Table S2 are also included below.

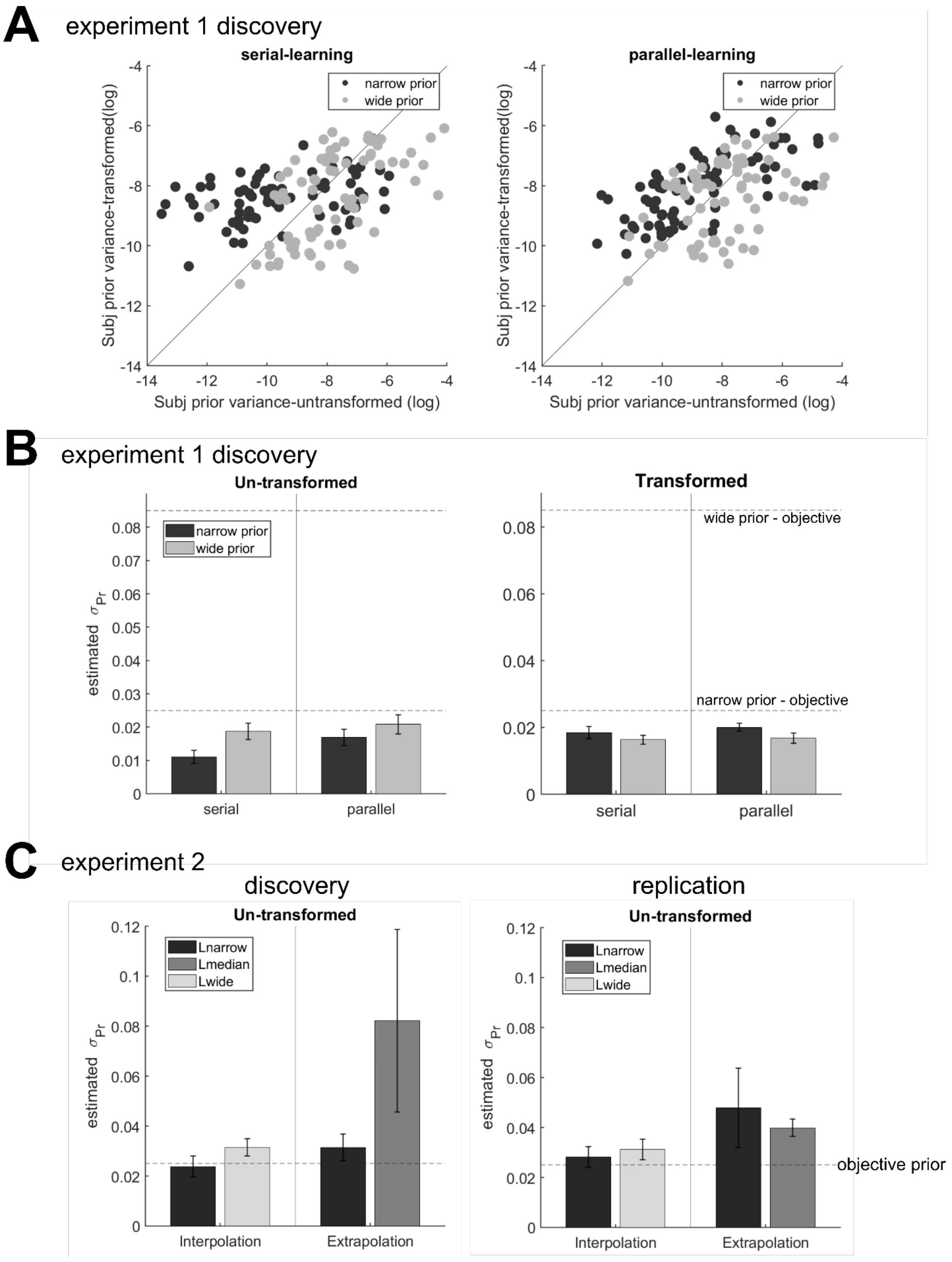

Figure S2 Subject-specific prior uncertainty (variance and standard deviation) A *Scatter plots of prior estimates, experiment 1 discovery set. Left panel: serial learning group; right panel: parallel learning group. The X-axis represents untransformed prior and the Y-axis represents logistic-transformed prior. The order of estimates among the same experimenter designed prior categories are largely maintained after transformation but the distributions of values are compressed, as expected.* ***B*** *Bar figures to compare the group means of un-transformed (left panel) and transformed (right panel) subject-specific priors of experiment 1, discovery set. Error bars are standard errors. Dash lines are the experimenter designed standard deviations of narrow (= 0.025) and wide (= 0.085) priors. Figures show that the order of different priors only maintained in the untransformed estimations. They also show that the numerical values are generally smaller than the experimenter designed values.* ***C*** *Bar figures to compare the group means of un-transformed subject-specific priors of the experiment 2. Dash lines are the experimenter designed standard deviations of the prior (= 0.025). Overestimation were observed, especially in the extrapolation group. Left panel: discovery set; right panel: validation set.*

participants N<br>included (excluded)	**slope**	**Subject-specific prior/predicted slope**	**transfer score**
**Experiment 1**			
Discovery - serial	48(0)	44(3)	43(1)
Discovery - parallel	47(1)	47(0)	43(4)
Validation - serial	78(2)	72(6)	67(5)
Validation - parallel	78(1)	75(3)	66(9)
**Experiment 2**			
Discovery - interpolation	35(0)	33(2)	28(5)
Discovery - extrapolation	40(0)	39(1)	28(11)
Validation - interpolation	82(0)	79(3)	68(11)
Validation - extrapolation	82(0)	73(9)	59(6)

Table S2 *Participant numbers after removal of outliers for grouped analyses of each measures including slope, predicted slope in the transfer phase and transfer score. Numbers in brackets are outliers who were excluded based on the criteria described in the supplementary methods.*

Later in the supplementary results ("**Untransformed versus logistic transformed subject-specific prior values**"), we argued why it is unlikely the interpolation-benefit effects we observed in our experiments are caused by these biases.

"While the logistic transformation kept the relation between different estimations of the same objective prior unchanged, the specific numeric values have changed (Figure S2 A). Importantly, this transformation also changed the relation between estimations of different prior sets (Figure S2 B). We concluded that it is preferable to use the un-transformed estimations (over the logistic transformed) in the computation of the transfer score.

There are cases where arbitrarily large un-transformed prior variances could bias transfer scores and the reasons are as follows. The denominator of the transfer score, [predicted slope$_{transfer}$-slope$_{learned}$], equals $[\frac{\sigma^2_{Psub}}{(\sigma^2_{Psub}+\sigma^2_{Lsub\_transfer})} - \frac{\sigma^2_{Psub}}{(\sigma^2_{Psub}+\sigma^2_{Lsub_{learned}})}]$. This term can be re-written as $\frac{(\sigma^2_{Lsub_{learned}}-\sigma^2_{Lsub_{transfer}})}{(\sigma^2_{Psub}+\sigma^2_{Lsub_{learned}}+\sigma^2_{Lsub\_transfer})}$, showing that the transfer score can overshoot beyond 1 when an estimated prior variance is arbitrarily larger than the likelihood uncertainty (as the denominator becomes arbitrarily small). This is an unavoidable measurement noise caused by using untransformed prior variances to compute the transfer score (but the reason to do so has been explained in the paragraph: supplementary Methods).

We further examined the effect of overestimations of priors (hence more likely showing a larger than 1 transfer score) in our results. Figure S2 B & C showed that overestimation of priors was most visible in the extrapolation condition in experiment 2. This is unsurprising given that slope values in the learning phase of the extrapolation condition were closer to 1. It is important to keep in mind that overestimations of

priors in the extrapolation group would have obscured, rather than cause, the observed difference of transfer scores between groups."

We have also included a paragraph in the discussion (manuscript L370) to address this limitation of transfer score in this study.

"One limitation of transfer score is that its accuracy is dependent on the accuracy of subject-specific prior estimates (eq. 4). When slopes are close to 1, there could be arbitrarily large prior variances (eq. 3) leading to unrealistic transfer scores. In this study we have made great efforts to reduce this negative effect (as described in supplementary: 'Handling arbitrarily large slopes' and 'Outlier participant exclusion criteria'). Importantly, by scrutinising the data (supplementary results and Figure S2), we showed that our transfer score patterns are unlikely to be subject to this limitation. However, we acknowledge that attrition of participants during the process of transfer score outlier exclusion is a shortcoming. This can potentially be improved in the future by choosing prior/likelihood combinations which are unlikely to produce large slope values."

11. Figure 3: You deal with this later with the transfer score, but you never say explicitly that the graphs in this figure pool participants together and thus the predicted slopes (the dashed and dotted lines) are based on the average (or median?) subject, and are not appropriate for individual subjects.

Following the reviewer's suggestion, we have integrated the figure of transfer scores of individual prior/likelihood combinations (original submission S1 Figure) into the main manuscript **Figure 3**. In the revision, we explicitly explained in the figure caption that transfer score data in the lower panels of **Figure 3. C & D** were merged from data of each prior/likelihood combination. Here we also include the updated figure and its caption.

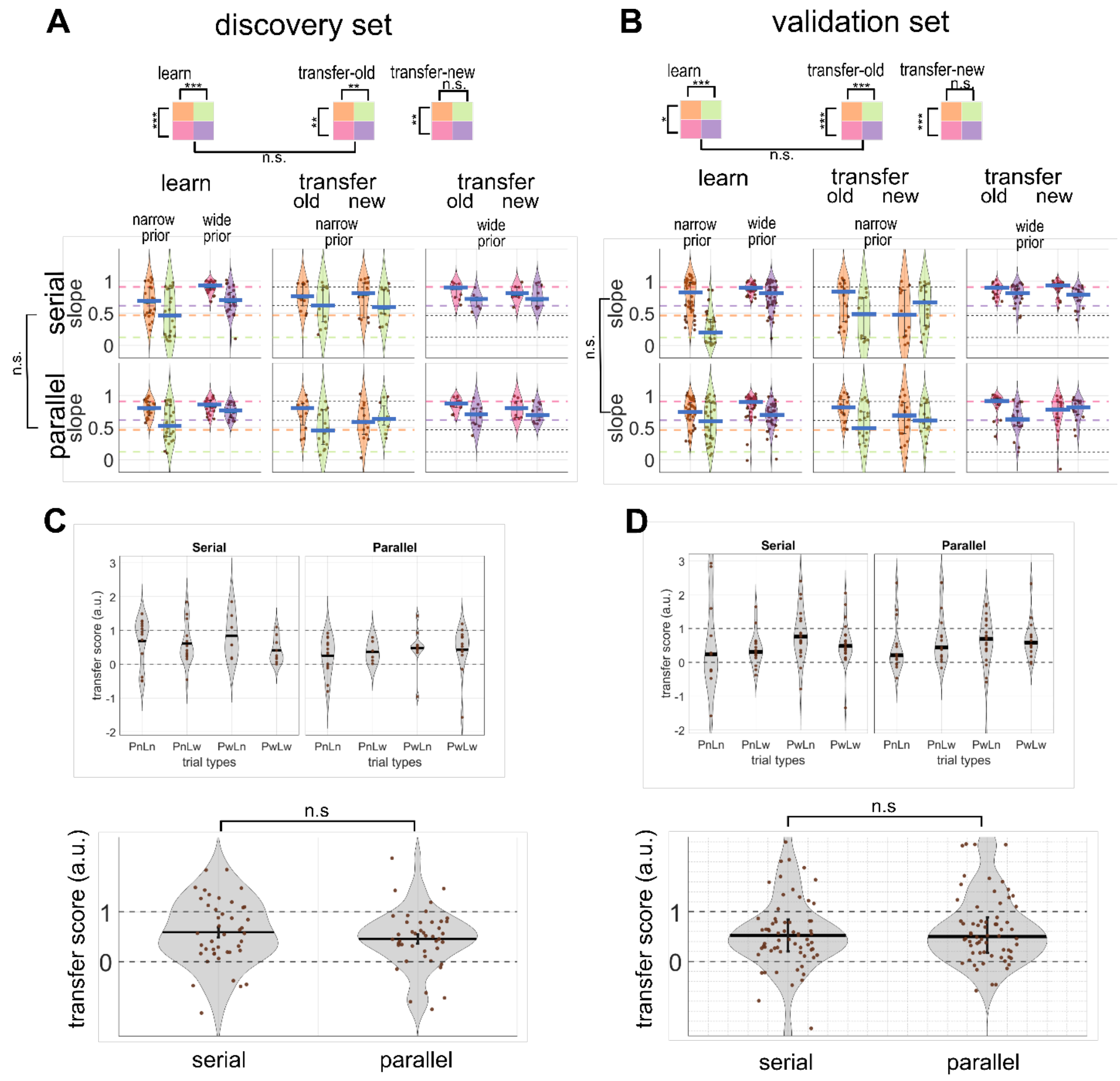

Fig 3. Slope and transfer score in the experiment 1. slope A Discovery & B Validation set *Violin plots show the distribution of slopes in the learning, transfer-old and transfer-new trials of experiment 1, separated by prior, likelihood and cognitive load group (upper serial, lower parallel). The central blue line shows the median. The error bar represents the interquartile range. Filled dots represent each participant. Each prior/likelihood combination is represented by orange PnLn, green PnLw, pink PwLn, and violet PwLw. Bayes-optimal values are presented as coloured dashed lines, with colours corresponding to prior/likelihood types. transfer score B Discovery & D validation sets. The distribution of transfer scores in the serial and parallel groups. The upper panels show distribution of transfer scores of each prior/likelihood combination, separated by cognitive load group. Lower panels merge data of different prior/likelihood combinations. The central black line and the error bar in the discovery set (Fig 3.B) represent the mean and standard error while central black line and the error bar in the validation set (Fig 3.D) represent the median and interquartile range. ** p<= .01; ***p<=.001; n.s. non-significant*

12. Figure 4: Inlets -> Insets

Corrected

13. S1 Figure: I'm not sure why S1 is kept out of the main text. It's the more appropriate analysis than the pooled-participant one in Figure 3.

As can be seen, we have moved the figure of transfer scores of individual prior/likelihood combinations (original submission S1 Figure) into the Figure 3 of the main manuscript.

Reviewer #2:

The authors present two studies aimed at finding out whether the computations supporting performance in a spatial estimation task are Bayesian. Knowledge transfer was the key criterion used to assess Bayesian computation—to the extent that information about previously learned priors and likelihoods could be used when combined in novel ways (Experiment 1) or when being tested on previously unexperienced likelihoods (Experiment 2), reflects the degree to which computation conforms to Bayesian expectations. Although performance was qualitatively Bayesian in all cases—combination of prior and likelihood information tended to be sub-optimal. Interestingly, combination of prior and a novel likelihood was better under "interpolation" conditions vs. "extrapolation". That is, when the novel variability in stimulus information was included within the range of previously experienced stimuli.

Overall, I found the manuscript to be interesting, and addressing an important question in a principled and sophisticated manner. I do have a few questions and concerns that I present below in no particular order.

Thank you for your kind appreciation of our work.

1 – One of the big issues for me is that the paper is framed in terms of asking whether or not computation is Bayesian, yet the question is addressed entirely within a Bayesian framework. To my mind, the approach taken by the authors cannot address the question of whether computation is Bayesian, but merely the extent to which performance can be approximated by Bayesian computation. This is an important distinction: even if performance is well-characterized by Bayesian computation, it does not follow that this is the underlying process. The same is true for any model-based description of behavior, Bayesian or not. This point is acknowledged by the authors to some extent in the discussion on pp. 19-20, but a more up-front declaration would be good to see.

Thank you for your suggestion, we had revised the second paragraph of the introduction to make the declaration more up-front (L58).

"We have previously (Lin & Garrido, 2022) **reviewed behaviours that fell short of Bayesian optimality but can still be better explained by Bayesian than heuristic strategies. We argued that examining how well performance matches Bayes-optimum is not effective in resolving whether computation conforms to Bayesian expectations, let alone understanding how the brain make probabilistic decision. Maloney and Mamassian** (Maloney & Mamassian, 2009) **have proposed a "generalization" criterion instead to evaluate whether the brain indeed makes inference in a Bayesian way. While people can learn a Bayes optimality policy by trial and error, these kinds of rote learners need to re-learn the policy whenever a change occurs in a learned task. As a result, they would likely fall short of the mark right after the change. On the contrary, a truly Bayesian agent that fully learns and flexibly accesses the components of the BDT will generalize efficiently by integrating new changes in the likelihood to any known priors** (Ma & Jazayeri, 2014)**. Generalization can be experimentally confirmed if a learned prior is transferred to novel conditions. This process has been coined as *Bayesian transfer*** (Maloney & Mamassian, 2009)**. We need to stress that *transfer* itself still concerns the computation strategies, meaning that it does not answer which algorithm the brain uses to approximate Bayesian computation. However, the pattern of a divergence from optimal transfer, can inform not only computation strategies but also guide our search for corresponding algorithms that suitably produce such strategies. For example, some studies have theorised how some**

algorithms implemented in the brain can compute the underlying statistical information of the world (Koblinger et al.,2021)**. These theories provide predictions about the patterns of generalization to unseen data beyond the context of past observations. There are also theories of Bayesian approximators that predict "generalization" behaviours which could only be observed within the context of past observations** (Dasgupta et al., 2020; Hasson et al., 2019)**. We can see how examining transfer is thus more informative than optimality matching in advancing our understanding of how the brain makes probabilistic decision-making."**

2 - Related to the above, I was struck by the absence of any comparison with an explicitly heuristic decision process. Although the authors state on page 19 that "There was no evidence supporting the use of a heuristic strategy in the experiment 1," later on they also mention "However, we cannot fully rule out that participants capitalised on the feature of the coin task to behave in a Bayesian fashion using a special case of heuristics." It is a little unclear to me how the authors were able to rule out—within limits—the use of heuristic decision strategies. My sense is that doing so would require specification of certain kinds of heuristic strategies and comparing human performance against an appropriately specified model. How would certain heuristic strategies perform in terms of the performance metrics (transfer score and optimality index) the authors presented?

We agree that there is a need for a comparison with heuristic decision process. Hence, we now constructed a linear regression model (which we described in the discussion in the previous edit of the manuscript) and a special form of the model, i.e. a likelihood-slope mapping model. We showed that linear models resulted in larger than 1 transfer scores in experiment 1 and insignificant transfer in the extrapolation groups of experiment 2 (supplementary results and **Figure S4**). Both features were at odds with our findings. Model comparison found both types of linear models fitted worse than the Bayesian model. Overall, our evidence did not support people used a linear mapping strategy performing the coin task.

Here we include the descriptions of the linear models in the Methods section (L593) and results of the linear models (L289)

Methods - **Linear regression models (L593) "Rather than computing slopes using Bayesian decision model, people can utilise a strategy of linearly mapping between estimated slopes and prior and likelihood reliability *(= inverse of variance).* The linear mapping can be expressed as the following equation: $slope = \beta_0 + \beta_1 \cdot \frac{1}{\sigma^2_{Psub}} + \beta_2 \cdot \frac{1}{\sigma^2_{Lsub}}$. For each participant, we first evaluated the coefficients $\{\beta_0, \beta_{1,} \beta_2\}$ using the learning phase data. We then plugged prior and likelihood variances in the transfer phase into the equation to predict transfer-phase slopes. In a special case of $\beta_1 = 0$, the model only accounts for a linear mapping between slope and likelihood reliability and we called it a likelihood-only model. Transfer scores predicted by these linear models are shown in the supplementary results and Figure S4. BIC was used to compare the fitness between Bayesian and linear models."**

Results - **Comparing with non-Bayesian models. (L289)**

"Comparing differences between modelled and measured transfer scores (Fig 6 C & D upper panels), we showed that the Bayesian model has the smallest difference to real world data in both experiments. Bayesian information criterion further showed evidence in favour of Bayesian model for both experiments (Fig 6 C & D lower panels). Simulated transfer scores of the linear regression, likelihood-only (Figure S4) and exemplar models (Figure S5) are presented in the supplementary materials."

Relevant supplementary information **including supplementary results and Figure S4** are also presented here

Supplementary results - **Linear regression and likelihood-only modelled transfer scores**

“We found that for experiment 1, the modelled transfer scores assuming both linear regression and likelihood-only models (Figure S4 A & B), were larger than 1 for both serial and parallel groups in the discovery and validation sets. This pattern was different from the experimental data. For experiment 2, the best fitted linear regression models had close to zero coefficients of priors, meaning the linear regression model in the experiment 2 was practically a likelihood-only model. This was proven by the fact that modelled slopes and transfer scores acquired from both models were identical. In Figure S4 we thus only present simulated results of the linear model because results of the likelihood-only model were the same. Similar to experimental observations, there was an interpolation benefit, i.e. a significantly higher transfer score in the interpolation than the extrapolation conditions (Figure S4 C discovery & D validation set). However, modelled transfer scores of the extrapolation group were not significantly different from 0 (discovery set *p* = 0.94, BF_{10} = 0.076, validation set *p* = 0.97, BF_{10} = 0. 052), which was not in line with experimental data.”

Figure S4 and its caption

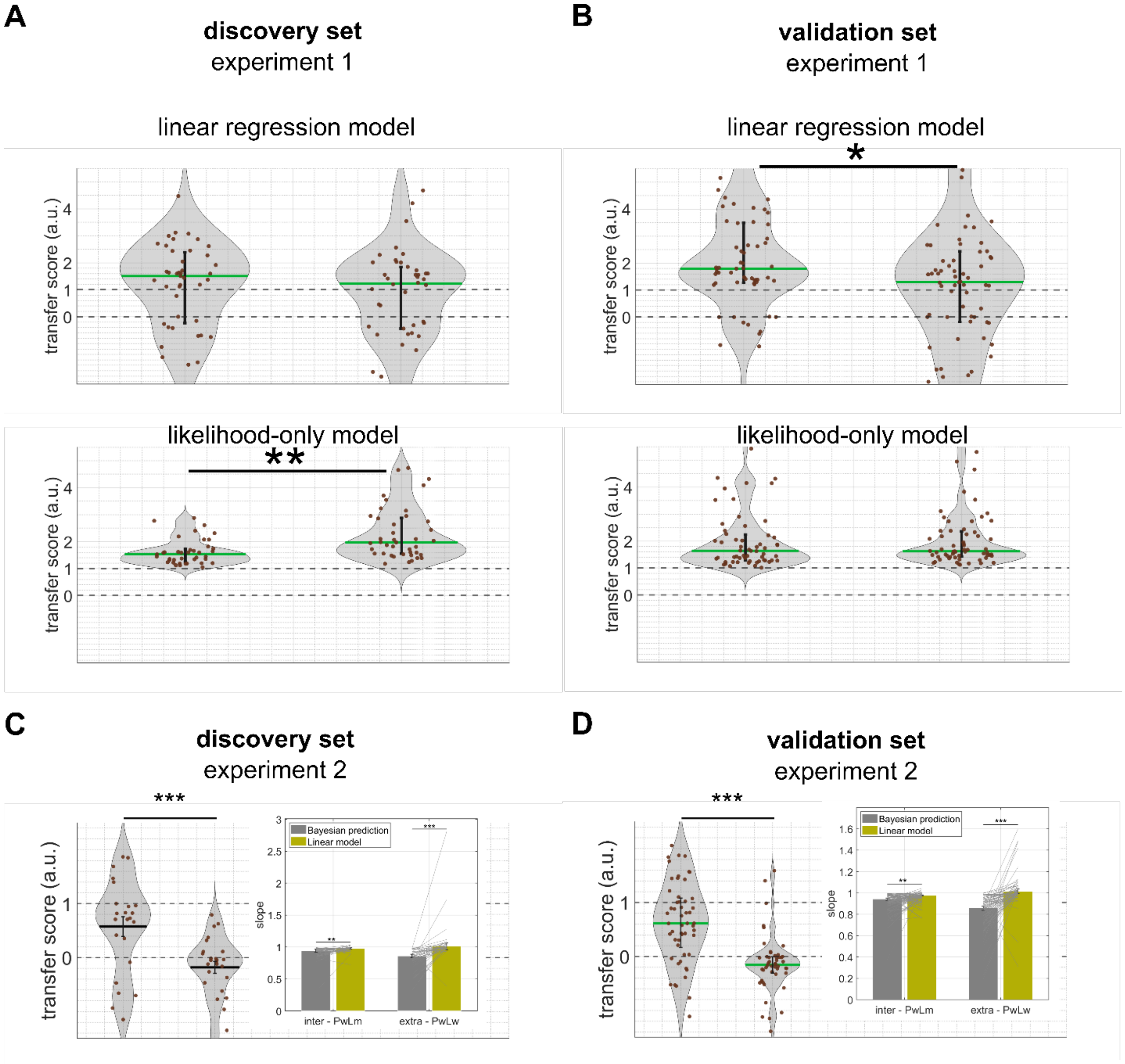

Figure S4 Simulated transfer scores of linear regression and likelihood-only models. ***A (experiment 1 discovery set) & B (experiment 1 validation set) show modelled transfer scores. The central green line is the median of each group, and the vertical bar is the interquartile range. Transfer scores are larger than 1 for both discovery and validation sets. C (experiment 2 discovery set) & D (experiment 2 validation set) show modelled transfer scores and insets compare slopes predicted by Bayesian model (grey bar) along with linearly modelled slopes (olive bar) of the transfer-new trials. Two important features are found: (1) transfer scores of the interpolation group are higher than those of the extrapolation group and (2) transfer scores of the extrapolation group are not significantly different from 0. *p<=.05, **p<=.01, ***p<=.001; n.s. non-significant***

Below are the Figure 6 and its caption, showing that Bayesian model (lowest in BIC) fitted better than linear models in both experiments.

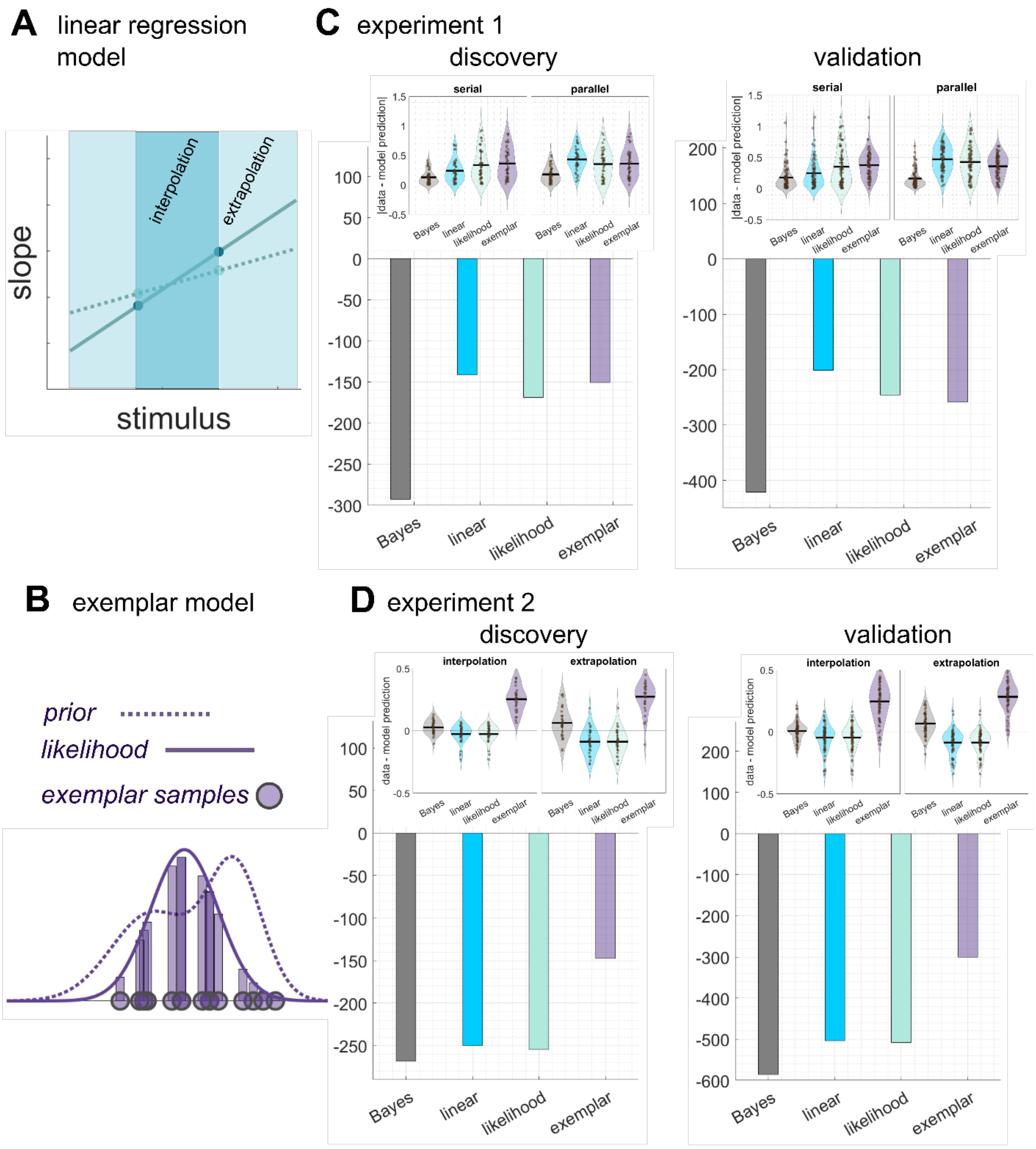

Fig 6 Computational models and comparison of model fitness with Bayesian decision model A schematic illustration of a linear regression model for the coin task. An optimal linear mapping between scatters of splashes and slopes (solid line) will produce optimal extrapolation even if no true generative process is represented. In reality, because of noisy slope estimations (dotted line), interpolation (darker green zone) would be closer to optimal than extrapolation (light green zone). B schematic illustration of an exemplar model for the coin task. Instead of representing the parameters of prior distribution, participants retrieved exemplar samples (violet circles) acquired from the learning phase. Samples are then weighted (bars representing weighting) by the gaussian likelihood of splashes to infer the coin position of a given trial. C (experiment 1) & D (experiment 2) Model comparison using Bayesian information criteria (BIC). Colour code: grey Bayesian model, blue linear regression model, green likelihood-only regression model, and violet exemplar model. The smaller BIC values indicate the better models. Insets showed differences between modelled and measured transfer scores.

3 – Reflecting on other potential approaches to the task that go beyond purely relying on the prior or likelihood, to what extent might people have been relying on exemplar memory to perform the task? Remembered instances of the true location of the coin, although sampled from the prior, would only gradually approximate the prior over time. This strategy would also intuitively produce an interpolation benefit—stored exemplars would cover the range of locations for interpolation transfer, but not extrapolation transfer.

We have constructed an exemplar model and examined how well the model fits the experimental findings.

In summary, we showed only when a sampled exemplar size was small (N = 5, see the definition of N below) we observed an interpolation benefit as have been seen in the experimental data. However, over-reliance on priors, which was at odds with the real data, was found in the modelled data (**Figure S5.A**). The interpolation benefit disappeared when sampled exemplar sizes increased (**Figure S5.D**). Importantly, Bayesian information criterion (BIC) showed that the exemplar model did not fit as well as the ideal Bayesian model. Together, we concluded evidence did not support participants utilised an exemplar model strategy to perform the task. All the contents related to the exemplar model can be found in the manuscript (L290, L365, L603 Figure 6.B,C & D) and supplementary materials (supplementary results and Figure S5) and below.

Firstly, the Methods (manuscript L603) which introduced the exemplar model:

“Exemplar model

We created an exemplar model of the coin task based on (Ross et al., 2014; Shi et al., 2010)**. Let us assume participants acquire the total past observed trials of coin locations during the learning phase as an exemplar set X^*. This means that priors are represented as exemplar memory instead of a probability distributions $p(x)$. Upon seeing the splashes in a trial *i*., *N* samples of exemplar $x^*$are drawn from X^*. Each drawn sample is then weighted by the splash distribution (i.e. likelihood distribution $p(\mu_{Li}, \sigma_L^2)$ where μ_{Li} is the centre of the splashes and σ_L^2 is the likelihood variance in trial i). We then take the normalised weighted sum of the values of sampled exemplars to get the posterior estimate $\dot{x}_i$ of the true coin location x_i. This exemplar model can be expressed as the equation below. Here the $f(x_j^*, X^*)$ is simply a function describing how samples are drawn from the exemplar set.**

$$\dot{x}_i = \frac{\sum_{j=1}^{n} f(x_j^*, X^*)\, p(x_j^* | \mu_i, \sigma_L)}{\sum_{j=1}^{n} p(x_j^* | \mu_L, \sigma_L)} \qquad (5)$$

In the coin task, we assumed samples are randomly drawn from all coin positions displayed in the learning phase. We implemented the model when the number of sampled exemplars size is small (*N*=5) and moderate (*N*=20). Modelled data of slopes and transfer score are shown in the supplementary results and Figure S5.”

Secondly, we presented modelled transfer scores, which are described in the supplementary results – exemplar model and **Figure S5**

“Simulated responses using the exemplar model

We used the exemplar model to simulate experimental responses. For experiment 2, modelled transfer scores of the discovery set were like experimental data when the sampled exemplar number was 5. That is, an interpolation benefit was observed (Figure S5.C). However, opposite to experimental findings, there was noticeable over-reliance on priors (Figure S5.A). Another feature dissimilar to the experimental data was that modelled transfer-new trials slopes were significantly higher than Bayesian model predictions in both interpolation and extrapolation groups (Figure S5. C&D). When we increased the sampled exemplar number to 20, over-reliance on prior became less obvious but the interpolation benefit vanished. Critically, transfer effects of both groups became insignificant (Figure S5.D). Modelled results of experiment 1 and

experiment 2 validation set had patterns highly like modelled results of experiment 2 discovery set, showing over reliance on priors and transfer score patterns similar to the real data in the case of small *N* but not moderate *N* (data not presented). Together with BIC results presented in the manuscript, evidence did not support that people utilise an exemplar model to perform the coin task."

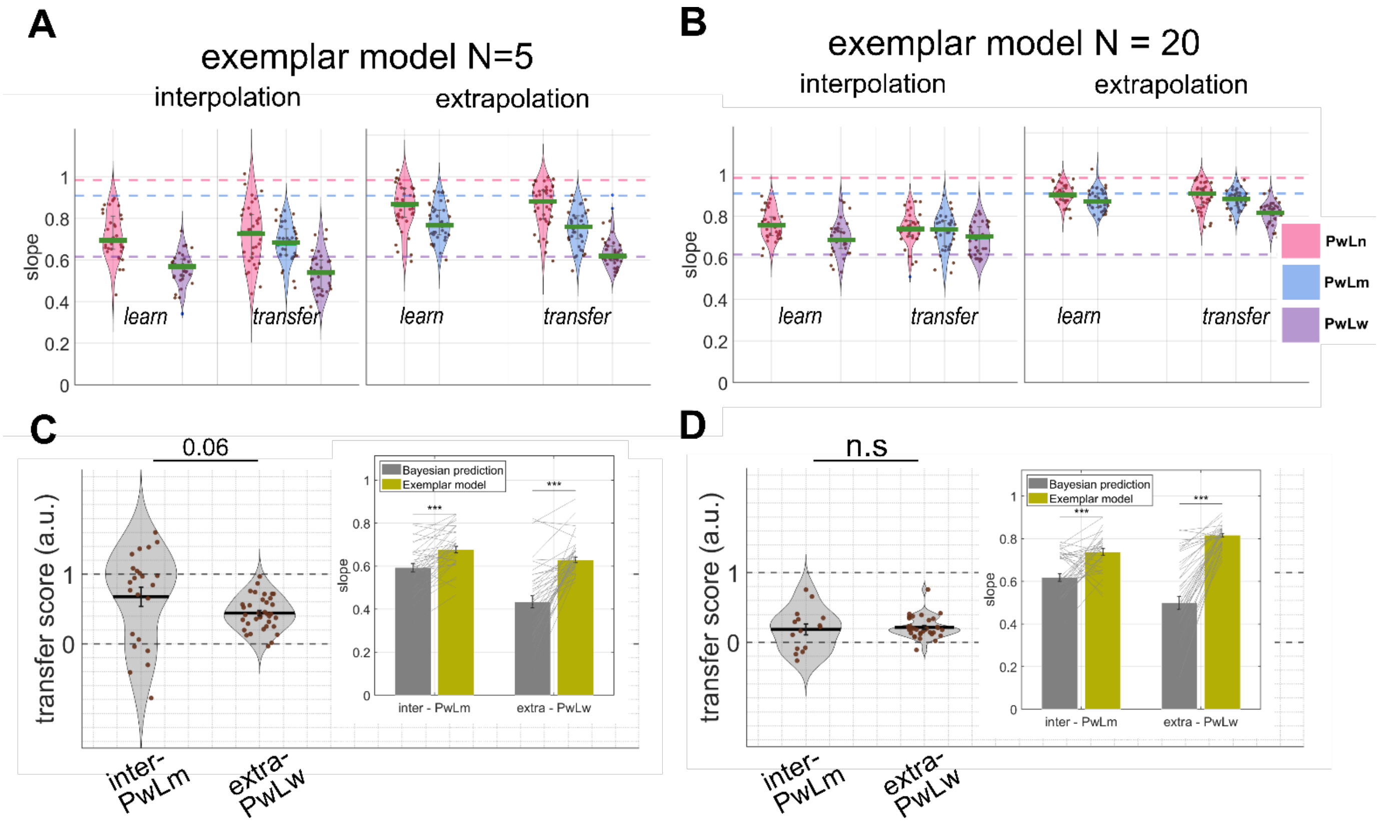

Figure S5 Exemplar modelled data of the experiment 2 discovery set. ***A (sampled exemplar size N = 5) & B (sampled exemplar size N = 20) show the distributions of modelled sensory weights and C (N = 5) & D (N = 20) show modelled transfer scores. Insets of C & D show predicted slope (grey bar) along with exemplar modelled slope (olive bar) of the transfer-new trials. ***p<=.001; n.s. non-significant***

Results of comparisons between Bayesian and exemplar models can be found in the manuscript L289:

"Comparing with non-Bayesian models

Comparing differences between modelled and measured transfer scores (Fig 6 C & D upper panels), we showed that Bayesian model has the smallest difference to real world data in both experiments. Bayesian information criterion further showed evidence in favour of Bayesian model for both experiments (Fig 6 C & D lower panels)."

Please also refer to our response to your comment 2, where we provided the updated **Figure.6**.

Discussion regarding our findings of comparison between the exemplar and Bayesian model in the manuscript (L364)

"…We also built an exemplar model to understand if people could also have used exemplar memory acquired from the learning phase for transfer and failed to find evidence support so…."

4 – I was a little confused about the computation of the transfer scores vs. the observed distribution of scores plotted in Figures 3 and 4. The measure is described as normalized, but because it can be positive/negative, so presumably it spans the interval [-1, 1]. I was unsure then how transfer scores greater

than 1 were obtained. My guess is that this would reflect a different kind of departure from optimality, overweighting the distribution of stimuli (likelihood) but discounting the prior?

To better explain the transfer score and how values greater than 1 could have been obtained, we revised the Methods-Transfer score as follows (L556):

"The transfer score compares the measured "change" of slopes against a predicted change of slopes computed by subject-specific estimates of prior and likelihood. In experiment 2, for each participant we would have acquired two measured slope changes using the two prior/likelihood combinations during the learning phase. We took their mean to calculate the transfer score of each participant. Note that transfer performance peaks at the value of one, (i.e. "Bayesian optimal" transfer score =1). A score equal to or smaller than zero means no transfer, and a score between zero and one indicates suboptimal transfer. Crucially a transfer score *systematically* larger than one is, from the Bayesian point of view, a deviation from optimum, not a "supra-optimal" transfer (e.g., excessive slope adjustments, examples can be seen in supplementary Figure S4). In our study there were also cases where even Bayesian computational strategies could show larger than 1 transfer score because of arbitrarily big subject-specific prior estimates (supplementary Figure S2). These undesirable artefacts were caused by noise introduced by the fitting procedure or measurement noise. We specifically devoted sections in the supplementary information (supplementary methods: 'handling arbitrarily large slopes' & 'outlier participant exclusion criteria') to explain how we minimised their effects in our data."

Additionally, we have removed "normalised" in the manuscript because we consider this term could have caused confusion.

5 – I struggled to follow the description of what was being depicted in Figure 6.

Thank you for your advice. We have revised Figure 6 and its caption to better suits the modelled data in the revision. Please also refer to our responses to your comment 2, where you can find the new figure 6.

We have also revised our description of the probabilistic recognition model in the manuscript (L366) to clarify the concept of it.

"**Together, it is possible that participants acquired a probabilistic recognition model linking contextualised sensory inputs to corresponding policies** (Dasgupta et al., 2020; Koblinger et al., 2021**. The model approximates a posterior where it has been covered by training data with a surrogate distribution. It would generalize well enough within, but not as well as outside past experience.**"

Reviewer #3:

The article evaluates human performance in tasks that can be modeled with Bayesian decision theory BDT. The elements of BDT are well known: prior, likelihood, loss function, etc. and whenever a human observer is given correct estimates of the objective prior, etc. we can compare human performance to ideal performance minimizing expected loss.

The authors claim that it has been experimentally established that human performance in Bayesian tasks is close to Bayes optimal but that there is still controversy over whether BDT is an accurate model of human performance. However, this controversy is justified. There is considerable experimental evidence dating to the 1950's that human observers in signal detection theory (Zhang & Maloney, 2012) distort the prior information given to them a phenomenon known as conservatism. Signal detection theory is a special case of BDT. This observed distortion precludes any claim that humans are Bayesian in all tasks. The authors should acknowledge that it is established that BDT is approximately correct for some tasks (the ones they cite) and not at all for others (Zhang & Maloney, 2012 reviews many examples of Bayesian failure due to distortion of probability). The answer to the question posed in the title is "Depends on the task "

Thank you for your comments. We agree with you that there are many behaviours that cannot be well characterised by an ideal Bayesian model. We consider the purpose of applying Bayesian framework is not to sample if tasks fit Bayesian optimal predictions or not. We also think the goal of utilise the Bayesian framework is to better inform how the brain process information. We have expressed the same viewpoints in a previously published review. Our writing in the introduction of this manuscript might have not been clear and thus caused misunderstandings. We have rephrased the first paragraph of the introduction (L48)

…Some evidence has shown that across various perceptual and cognitive tasks (Alais & Burr, 2004; Battaglia et al., 2003; Ernst & Banks, 2002; Griffiths & Tenenbaum, 2006; Knill & Whitman Richards., 1996; Körding & Wolpert, 2004; Navalpakkam et al., 2010) **behaviours are close to Bayes-optimal. However, there are behaviours which are qualitatively Bayesian (i.e., following the reliability-based weighting principle) but fall short of Bayes-optimality** (review see Denison et al 2018)**, hence casting doubt upon the ideal Bayesian-observer theory. Studies have also shown cases where neurotypical** (Zhang & Maloney, 2012) **or neurodivergent populations** (Adams et al., 2021; Jardri & Denève, 2013) **can fail to represent the true probability distribution of the world. These findings brought into questions whether the brain computes behaviours using the Bayes rule** (Bowers & Davis, 2012; Gardner, 2019; Jones & Love, 2011)**…**

But the major thrust of the paper is not to evaluate the Bayesian computation but rather to test whether the brain treats different priors as modules that can be extracted from any particular task and transferred to another task. Maloney & Mamassian (2009) referred to this test as a transfer test. In detail: suppose an observers learn a prior and a loss function in one Bayesian task and a different prior and a different loss function in a second task. The learning process may be slow if the tasks are complex. Can the observer then perform a novel task with the prior from the first task and the loss function from the second without further learning?

Maloney & Mamassian point out the work of Trommershauser, Maloney & Landy contains such transfer tasks which the observer successfully negotiates. You might cite Maloney & Mamassian earlier in the article where you introduce their test.

The central part of the article is a series of transfer tests and the experimental work an the results of these tests are very well done. Measuring degree of transfer is elegant. My main criticism of the article is that the conceptual framework is incorrect or misleading but the experimental work reported is of significant value.

Thank you for appreciating the values of our work. We are also grateful for your constructive suggestions. Taking the suggestions, we now cited Maloney & Mamassian's work earlier in the 2nd paragraph of the introduction (L58) and also have made substantial changes to the paragraph to better state our central idea: testing Bayesian transfer to better understand strategies that the human brain utilises in decision-making:

"**We have previously (Lin & Garrido, 2022) reviewed behaviours that fell short of Bayesian optimality but can still be better explained by Bayesian than heuristic strategies. We argued that examining how well performance matches Bayes-optimum is not effective in resolving whether computation conforms to Bayesian expectations, let alone understanding how the brain make probabilistic decision. Maloney and Mamassian (Maloney & Mamassian, 2009) have proposed using a "generalization" criterion instead to evaluate whether the brain indeed makes inference in a Bayesian way. While people can learn a Bayes optimality policy by trial and error, these kinds of rote learners need to re-learn the policy whenever a change occurs in a learned task. As a result, they would likely fall short of the mark right after the change. On the contrary, a truly Bayesian agent that fully learns and flexibly accesses the components of the BDT will generalize efficiently by integrating new changes in the likelihood to any known priors (Ma & Jazayeri, 2014). Generalization can be experimentally confirmed if a learned prior is transferred to novel conditions. This process has been coined as *Bayesian transfer* (Maloney & Mamassian, 2009). We need to stress that *transfer* itself still concerns the computation strategies, meaning that it does not answer which algorithm the brain uses to approximate Bayesian computation. However, the pattern of a divergence from optimal transfer, can inform not only computation strategies but also guide our search for corresponding algorithms that suitably produce such strategies. For example, some studies have theorised how some**

algorithms implemented in the brain can compute the underlying statistical information of the world (Koblinger et al., 2021). These theories provide predictions about the patterns of generalization to unseen data beyond the context of past observations. There are also theories of Bayesian approximators that predict "generalization" behaviours which could only be observed within the context of past observations (Dasgupta et al., 2020; Hasson et al., 2019). We can see how examining transfer is thus more informative than optimality matching in advancing our understanding of how the brain makes probabilistic decision-making."

Regarding the question of testing generalisation by performing a novel task with the prior from the first task and the loss function from the second without further learning, our answers are as follows. While this was the main example that Maloney & Mamassian mentioned in the 2009 paper, they also agreed that 'transfer' can be tested by substituting any of the components in the Bayesian decision theory (prior, likelihood or cost function). Here we chose to mainly investigate the effect of prior/likelihood substitution.

Regarding concerns about our conceptual framework, we believe that our explicit acknowledgement of the limitations of the explanatory values of Bayesian decision theory in general and optimality more specifically are in line with the reviewer's point. We hope that by revising the first and second paragraphs of the introduction, as presented here, we have made our research question clearer. That is, we believe that testing transfer, as compared to matching behaviours to 'Bayes optimum', is more informative for understanding the underlying strategies of decision making. However, a major shortcoming of previous reports on Bayesian transfer is that they still confine to the "Bayesian optimum" idea and we proposed a quantifiable definition which can better define transfer.

Have the authors made all data and (if applicable) computational code underlying the findings in their manuscript fully available?

The PLOS Data policy requires authors to make all data and code underlying the findings described in their manuscript fully available without restriction, with rare exception (please refer to the Data Availability Statement in the manuscript PDF file). The data and code should be provided as part of the manuscript or its supporting information, or deposited to a public repository. For example, in addition to summary statistics, the data points behind means, medians and variance measures should be available. If there are restrictions on publicly sharing data or code —e.g. participant privacy or use of data from a third party—those must be specified.

Reviewer #1: Yes

Reviewer #2: **No:** Details will be made available upon acceptance, I presume.

Reviewer #3: Yes

Data will be available on the openscience framework https://osf.io/

Code will be available on GitHub https://github.com/

PLOS authors have the option to publish the peer review history of their article (what does this mean?). If published, this will include your full peer review and any attached files.

If you choose "no", your identity will remain anonymous but your review may still be made public. **Do you**

want your identity to be public for this peer review? For information about this choice, including consent withdrawal, please see our Privacy Policy.

Reviewer #1: **Yes:** Michael S Landy

Reviewer #2: No

Reviewer #3: No

References

Acerbi, L., Vijayakumar, S., & Wolpert, D. M. (2014). On the Origins of Suboptimality in Human Probabilistic Inference. *PLoS Computational Biology*, *10*(6), e1003661. https://doi.org/10.1371/journal.pcbi.1003661

Dasgupta, I., Schulz, E., Tenenbaum, J. B., & Gershman, S. J. (2020). A theory of learning to infer. *Psychological Review*, *127*(3), 412–441. https://doi.org/10.1037/rev0000178

Hasson, U., Nastase, S. A., & Goldstein, A. (2019). Perspective Direct Fit to Nature : An Evolutionary Perspective on Biological and Artificial Neural Networks. *Neuron*, *105*(3), 416–434.

Koblinger, Á., Fiser, J., & Lengyel, M. (2021). Representations of uncertainty: where art thou? *Current Opinion in Behavioral Sciences*, *38*, 150–162. https://doi.org/10.1016/j.cobeha.2021.03.009

Lin, C.-H. S., & Garrido, M. I. (2022). Towards a cross-level understanding of Bayesian inference in the brain. *Neuroscience & Biobehavioral Reviews*, *137*(March), 104649. https://doi.org/10.1016/j.neubiorev.2022.104649

Ma, W. J., & Jazayeri, M. (2014). Neural Coding of Uncertainty and Probability. *Annual Review of Neuroscience*, *37*, 205–220. https://doi.org/10.1146/annurev-neuro-071013-014017

Maloney, L. T., & Mamassian, P. (2009). Bayesian decision theory as a model of human visual perception: Testing Bayesian transfer. *Visual Neuroscience*, *26*, 147–155. https://doi.org/10.1017/S0952523808080905

Sato, Y., & Körding, K. P. (2014). How much to trust the senses: learning likelihoods. *Journal of Vision*, *14*(13), 1–13. https://doi.org/10.1167/14.13.13.doi

Vilares, I., Howard, J. D., Fernandes, H. L., Gottfried, J. A., & Kording, K. P. (2012). Differential representations of prior and likelihood uncertainty in the human brain. *Current Biology*, *22*, 1641–1648. https://doi.org/10.1016/j.cub.2012.07.010
