# Supplementary material for: Are we really Bayesian? Probabilistic inference shows sub-optimal knowledge transfer
Source: PLoS Comput Biol. 2024 Jan 8;20(1):e1011769. doi: 10.1371/journal.pcbi.1011769 (PMC10798629; doi:10.1371/journal.pcbi.1011769)
Supplement: Attachment — Submitted filename: Response to reviewers_revision2.pdf [file pcbi.1011769.s010.pdf]

Are we really Bayesian? Probabilistic inference shows sub-optimal knowledge transfer

Response to reviewers

We again thank the reviewers for their constructive comments. In the following, we respond to each point of these comments with texts in blue colour. We also marked changes to the manuscript text in **bold font** and provided their line numbers in the updated manuscript.

Reviewer #1:

Reviewer: Michael Landy

This revision is extremely thorough in response to my and the other reviewers' comments. My only comment is somewhat of a repeat. There's a model used to provide for a predicted subject-specific transfer-phase slope. Now, if you double the prior variance and double the likelihood "variance" (well, the measurement distribution variance), the predicted slope doesn't change. Thus, to get a predicted slope change, you kind of want to nail down one or the other, then learn the other one from condition A, then predict condition B. The authors do this as represented by Eq. 2.

I still find that equation odd as a model of subjective likelihood variance. In the likelihood-only task, the proper thing to do (and hopefully what participants are doing) is point at the centroid (since that's the max-likelihood position). If a participant fails to hit the centroid, the error will be some combination of dot-location-measurement error, centroid-computation error, and response/motor error. This variance will be independent of the error of centroid relative to "true" coin location (that error comes from the independent sampling of the splash). Thus, Eq. 2 calculates the sum of the participant's pointing error variance and the true likelihood variance. Why is this a reasonable way of estimating "subject likelihood variance"? There's nothing subjective in this. Usually, one gets at subjective variance by looking at, e.g., the slope, overtraining the prior, and attributing non-optimal slope as an indication of subjective variance. Thus, in others' papers, the only way to get subjective variance is to assume Bayesian optimal calculation with distorted inputs (e.g., incorrect subjective likelihood variance). So, a large stretch of your paper is based on a model of subjective likelihood variance that, frankly, I don't accept. I'm not sure what to do about that.

Otherwise, all good!

Thank you for your comment and we are pleased to know that you appreciate our thorough revision. We acknowledge that σ_{Li}^2 , i.e. the variance computed by the eq.3 (eq.2 of the last revision), accounts for more than perceptual noise. σ_{Li}^2 measures how precisely people can estimate the true likelihood centroid and is hence used as a proxy of how reliable likelihood information is to a person's location judgement. Hence, it is in that sense subject-specific. We agree that this measure is a combination of perceptual, computation and motor noise. However, evidence has shown that this measure in fact explains better how people decided sensory weights in the coin task (Kiryakova et al., 2020). For these reasons we think it is more appropriate to replace experimenter-imposed likelihood variances with σ_{Li}^2. We have revised the subsection - **Predicted slope in the transfer phase based on proxies for subject-specific prior and likelihood variances** (L535). We addressed the difference between σ_{Li}^2 (eq.2 & 3) and σ_L^2 (eq.1) and presented our argument of using σ_{Li}^2 . We also gave a step-by-step thorough explanation for why and how we acquire the predicted transfer slope based on these proxies to ensure we expressed our case clearly to readers.

L537: "Numerous studies using the coin task and similar paradigms (Bejjanki et al., 2016; Berniker et al., 2010; Randeniya et al., 2021; Sato & Körding, 2014; Vilares et al., 2012; Vilares & Kording, 2017)**, consistently**

demonstrated that people integrate prior and likelihood information in a qualitatively Bayesian fashion. However, empirical slope values are often different from optimal Bayesian values. This difference increases the measurement noise of slope values in the transfer phase. Therefore, a slope value which deviates from Bayes optimal during transfer may either be a manifestation of suboptimal Bayesian behaviours (Lin & Garrido, 2022)**, or a result of a non-Bayesian strategy. Simply measuring slopes cannot discriminate the two, so it is an ineffective way of determining transfer. Instead, we postulate a measure inferring knowledge transfer (as defined in eq.5 in the next section: transfer score) that relies on proxies for subject-specific prior and likelihood variances as described below.**

Studies have shown that instead of estimating coin locations based on experimenter-imposed prior and likelihood variability, people's judgments of the coin location are more likely weighted by the participants' estimated variability of likelihood and prior (i.e. perceived errors to them) (Kiryakova et al., 2020; Randeniya et al., 2021)**. To express this concept mathematically, we changed (eq.1) to**

$$\dot{X}_{est} = \frac{\sigma_{Li}^2}{\sigma_{Pi}^2 + \sigma_{Li}^2}\mu_P + \frac{\sigma_{Pi}^2}{\sigma_{Pi}^2 + \sigma_{Li}^2}\mu_L \qquad (2)$$

, which shows that a participant's estimated position $\dot{X}_{est}$ is again a weighted average of prior mean μ_P and likelihood mean μ_L. However, in contrast to eq.1, weights are based on estimated subject-specific behavioural precision for the prior and likelihood. Specifically, σ_{Pi}^2 evaluates how precisely participants can locate a hidden coin in the absence of splashes and σ_{Li}^2 , a proxy for subject-specific likelihood, evaluates the participants' precision of estimated centres of likelihood. Importantly, we can approximate σ_{Pi}^2 and σ_{Li}^2 from individuals' likelihood-only task and learning phase data to predict transfer-phase slopes and then compare against real data in the transfer phase. In the following, we explain how we did so step-by-step.

Firstly, we calculated σ_{Li}^2, the variance of estimates of the mean (μ_{Lest}) relative to the true mean of the splashes (μ_L) in the likelihood-only task (Randeniya et al., 2021; Goodwin et al., 2023; Goodwin et al., in press)

$$\sigma_{Li}^2 = \frac{\sum(\mu_{Lest} - \mu_L)^2}{n_{Trials}} \qquad (3)$$

The number of trials (n_{Trials}) for each likelihood condition was 40 in the experiment 1 and 30 in the experiment 2. Note that σ_{Li}^2 not only represents sensory precision but also captures a combination of errors of perception (dot-location-measurement), computation (centroid-computation), and action (response/motor). Importantly, this proxy for subjective likelihood variance has been shown to explain slope data better than using experimenter-imposed (by design) likelihood variance (Kiryakova et al., 2020)**. Secondly, σ_{Pi}^2 is computed as a combination of σ_{Li}^2 and sensory weight, which is derived by rearranging the equation ($slope = \frac{\sigma_{Pi}^2}{\sigma_{Pi}^2+\sigma_{Li}^2}$) as follows** (Randeniya et al., 2021; Goodwin et al., 2023; Goodwin et al., in press)

$$\sigma_{Pi}^2 = \frac{\sigma_{Li}^2 * slope}{(1 - slope)} \qquad (4)$$

Thirdly, assuming σ_{Pi}^2 and σ_{Li}^2 were stable between learning and transfer phases, for each participant the σ_{Pi}^2 and σ_{Li}^2 of transfer-new trials were then plugged back into the equation ($slope = \frac{\sigma_{Pi}^2}{\sigma_{Pi}^2+\sigma_{Li}^2}$) to acquire a predicted transfer-phase slope."

We also made a minor change in the description of the likelihood-only task to ensure we communicated the purpose of administering this task correctly.

L461: "We also administered a likelihood-only task to measure how good participants were at locating the centroids of dot clouds at each level of likelihood uncertainty (σ_L = 0.0024, 0.06 and 0.15) in the absence of prior information."

Reviewer #2:

I thank the authors for their thoughtful and comprehensive responses to my earlier concerns. Direct comparison of the Bayesian model with the linear regression and exemplar-based heuristic models has substantially improved the strength of the claims made by the authors. The more detailed discussion of exactly what we can glean from this research (e.g., going beyond simply comparing performance against a Bayesian standard; identifying cases of qualitatively Bayesian behavior and diagnosing where processing deviates from optimality) has also improved the clarity of the manuscript. I have no further issues or concerns, and think the manuscript can be accepted in its revised form.

We thank the reviewer for approving our efforts in addressing those concerns of the previous submission.

References:

Bejjanki, V. R., Knill, D. C., & Aslin, R. N. (2016). Learning and inference using complex generative models in a spatial localization task. *Journal of Vision*, *16*(5), 1–13. https://doi.org/10.1167/16.5.9

Berniker, M., Voss, M., & Kording, K. (2010). Learning priors for bayesian computations in the nervous system. *PLoS ONE*, *5*(9), 1–9. https://doi.org/10.1371/journal.pone.0012686

Goodwin, I., Hester, R., & Garrido, M. I. (2023). *Temporal Stability of Bayesian Belief Updating in Perceptual Decision Making*. https://doi.org/10.31234/osf.io/wj8xh

Goodwin, I., Kugel, J., Hester, R., & Garrido, M. I. (2023). Bayesian Accounts of Perceptual Decisions in the Nonclinical Continuum of Psychosis: Greater Imprecision in Both Top-down and Bottom-up Processes. *PLoS Computational Biology*. https://doi.org/10.1371/journal.pcbi.1011670

Kiryakova, R. K., Aston, S., Beierholm, U. R., & Nardini, M. (2020). Bayesian transfer in a complex spatial localization task. *Journal of Vision*, *20*(6), 17. https://doi.org/10.1167/jov.20.6.17

Lin, C.-H. S., & Garrido, M. I. (2022). Towards a cross-level understanding of Bayesian inference in the brain. *Neuroscience & Biobehavioral Reviews*, *137*(March), 104649. https://doi.org/10.1016/j.neubiorev.2022.104649

Randeniya, R., Vilares, I., Mattingley, J. B., & Garrido, M. I. (2021). Reduced Context Updating but Intact Visual Priors in Autism. *Computational Psychiatry*, *5*(1), 140. https://doi.org/10.5334/cpsy.69

Sato, Y., & Körding, K. P. (2014). How much to trust the senses: learning likelihoods. *Journal of Vision*, *14*(13), 1–13. https://doi.org/10.1167/14.13.13.doi

Vilares, I., Howard, J. D., Fernandes, H. L., Gottfried, J. A., & Kording, K. P. (2012). Differential representations of prior and likelihood uncertainty in the human brain. *Current Biology*, *22*(18), 1641–1648. https://doi.org/10.1016/j.cub.2012.07.010

Vilares, I., & Kording, K. P. (2017). Dopaminergic medication increases reliance on current information in Parkinson's disease. *Nature Human Behaviour*, *1*(8), 1–7. https://doi.org/10.1038/s41562-017-0129
